# Regulation of senescence escape by TSP1 and CD47 following chemotherapy treatment

**DOI:** 10.1038/s41419-019-1406-7

**Published:** 2019-02-27

**Authors:** Jordan Guillon, Coralie Petit, Marie Moreau, Bertrand Toutain, Cécile Henry, Henry Roché, Nathalie Bonichon-Lamichhane, Jean Paul Salmon, Jérôme Lemonnier, Mario Campone, Véronique Verrièle, Eric Lelièvre, Catherine Guette, Olivier Coqueret

**Affiliations:** 1 Paul Papin ICO Cancer Center, CRCINA, INSERM, Université de Nantes, Université d’Angers, Angers, France; 2 Institut Universitaire du Cancer, Toulouse, France; 3 Clinique Tivoli, Bordeaux, France; 4 Centre Hospitalier PELTZER-LA TOURELLE, Verviers, Belgium; 5 R&D UNICANCER, UCBG Paris, Paris, France; 6 SIRIC ILIAD, Nantes, Angers, France

## Abstract

Senescence is a tumor-suppressive mechanism induced by telomere shortening, oncogenes, or chemotherapy treatment. Although it is clear that this suppressive pathway leads to a permanent arrest in primary cells, this might not be the case in cancer cells that have inactivated their suppressive pathways. We have recently shown that subpopulations of cells can escape chemotherapy-mediated senescence and emerge as more transformed cells that induce tumor formation, resist anoikis, and are more invasive. In this study, we characterized this emergence and showed that senescent cells favor tumor growth and metastasis, in vitro and in vivo. Senescence escape was regulated by secreted proteins produced during emergence. Among these, we identified thrombospondin-1 (TSP1), a protein produced by senescent cells that prevented senescence escape. Using SWATH quantitative proteomic analysis, we found that TSP1 can be detected in the serum of patients suffering from triple-negative breast cancer and that its low expression was associated with treatment failure. The results also indicate that senescence escape is explained by the emergence of CD47^low^ cells that express a reduced level of CD47, the TSP1 receptor. The results show that CD47 expression is regulated by p21waf1. The cell cycle inhibitor was sufficient to maintain senescence since its downregulation in senescent cells increased cell emergence. This leads to the upregulation of Myc, which then binds to the CD47 promoter to repress its expression, allowing the generation of CD47^low^ cells that escape the suppressive arrest. Altogether, these results uncovered a new function for TSP1 and CD47 in the control of chemotherapy-mediated senescence.

## Introduction

Chemotherapy-induced senescence (CIS) is a tumor-suppressive mechanism that occurs in vitro and in vivo and has been detected in tumor samples following neoadjuvant chemotherapy^1,2^. Although arrested, senescent cells communicate with neighboring clones through soluble factors known as the senescence-associated secretory phenotype (SASP)^3–5^. This secretome prevents the abnormal proliferation of bystander clones^6^, attracts immune cells^7,8^ but it can also exert oncogenic functions and induces chemotherapy resistance^9–11^. In addition, the clearance of senescent cells increases the life span and reduces carcinogenesis^12^. Thus, senescence can also alter the microenvironment and favor tumor progression and this questions its clinical value as compared with apoptosis^13^.

In response to treatment, it is also unclear whether CIS is always irreversible. By definition, a tumor-suppressive mechanism has to be inactivated during cancer progression. Advanced cancer cells can still activate the CIS program but this cannot lead to a complete arrest if suppressive pathways have been inhibited during cell transformation. To understand these adaptive mechanisms, we have developed models of senescence escape, either in response to oncogenes^14,15^ or to chemotherapy^16–19^. We reported that subpopulations of cells escape senescence to generate emergent cells that are more transformed and resist anoikis.

We now extend these observations and show that emergent cells produce secreted proteins that regulate CIS escape. The deleterious effect of senescent cells was confirmed in mice, increasing tumor growth and metastasis. We identified thrombospondin-1 (TSP1) as a protein secreted by senescent cells which maintains the proliferative arrest. Using quantitative proteomics, we show that a low TSP1 level is predictive of chemotherapy failure in patients suffering from triple-negative breast cancer. Our results also describe new functions for CD47, one of the TSP1 receptors. Senescence escape is explained by the appearance of persistent cells that express reduced levels of CD47 and p21waf1. The results indicate that p21waf1 downregulation increases Myc expression, which then binds to the CD47 promoter to repress its activity. This downregulates the surface expression of the receptor and generates CD47^low^ cells that escape senescence.

Altogether, these results indicate that some subpopulations can escape chemotherapy-induced senescence. This suppression is normally maintained by a high expression of p21waf1 that prevents Myc activation and the generation of CD47^low^ cells. We propose that CD47 targeting should be applied with caution when used in combination with genotoxic treatments.

## Results

### Senescence escape in response to genotoxic treatment

**Fig. 1:**
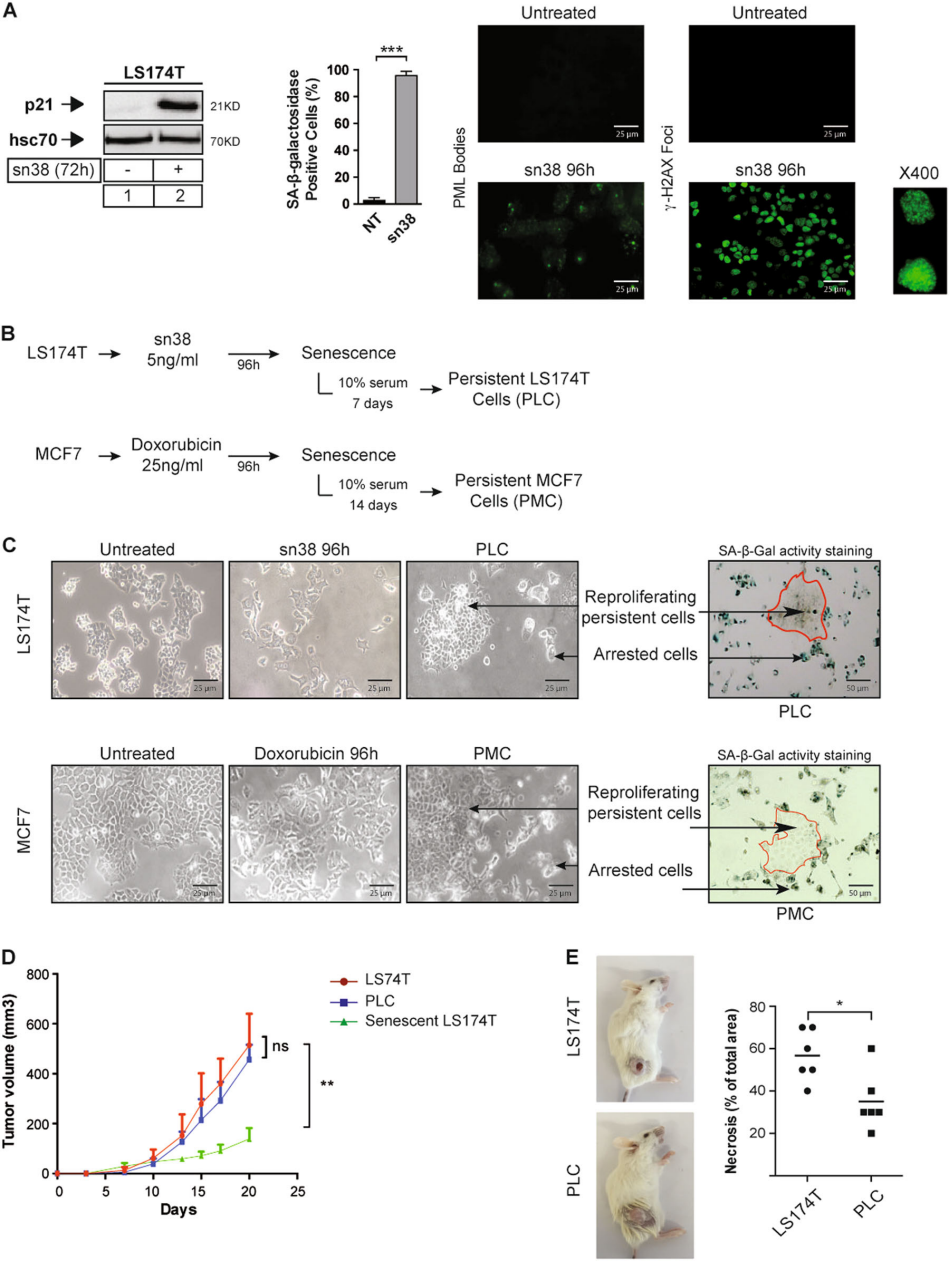
Colorectal and breast cancer cell lines escape chemotherapy-mediated senescence. **a** Following sn38 treatment (5 ng/ml), senescence was detected by the evaluation of p21waf1 expression, SA-β-galactosidase, PML bodies, and ɣ-H2AX staining (*n* = 3 +/−sd, Mann–Whitney test, *** = *p* < 0.001). **b** After treatment, LS174T cells were washed and stimulated with 10% FBS for 7 days to reinduce cell growth. Persistent MCF7 cells (PMC) were generated using the same protocol following doxorubicin treatment. **c** Images illustrating persistence using colorectal (top) or breast (bottom) cells. SA-β-galactosidase activity staining shows PLC heterogeneity and the presence of proliferating cells (white cells, named PLD) together with senescent cells (blue cells, named PLS). **d** In vivo evaluation of tumor formation by parental LS174T cells, senescent cells, or PLCs. Senescent and emergent cells were generated as described above. Cells were injected subcutaneously in NOD/SCID mice (five mice were used per condition in each experiment, two-way Anova with a Bonferroni’s multiple comparison test: ns = *p* > 0.05. ** = *p* < 0.01). **e** Quantification of necrosis in tumors arising from parental or PLC cells (*n* = 6, Mann–Whitney test, * = *p* < 0.05, see also supplementary Figure 1)

We first confirmed our observations^16,17^, showing that genotoxic treatments induce senescence. p21waf1 was upregulated and CIS was confirmed using SA-β-galactosidase, PML bodies, and ɣ-H2AX staining in LS174T colorectal cells and MCF7 breast cells (Fig. 1a, supplementary Figure 1). We recently reported that subpopulations of colorectal cells can adapt to CIS and resume proliferation^14–17^. Escape from senescence leads to the emergence of more transformed cells that we have named PLC (persistent LS174T cells, Fig. 1b, see Materials and Methods for a summary of the names of all subpopulations). After 7 days, the PLC population is heterogeneous and composed of around 60–70% senescent cells (named PLS—persistent LS174T senescent cells) and 30–40% of proliferating cells (named PLD—persistent LS174T dividing cells). SA-β-galactosidase staining illustrating this heterogeneity is shown Fig. 1c. Persistence was also observed using MCF7 cells (Fig. 1c). We have already shown that this is not due to the presence of a resistant clone in parental cells^17,19^. We have described that emergent cells are more aggressive than parental cells, they induce tumor formation in mice and resist anoikis^14,16,17^. This was confirmed in the present study: in immunocompromised mice, PLC formed tumors to the same extent as parental cells despite the fact that they were mainly arrested and composed of 70% senescent cells (Fig. 1d). Senescent cells alone grew less efficiently, which was expected if some cells also escape senescence in mice. Tumors arising from PLC growth presented fewer necrotic cells (Fig. 1e, supplementary Figure 2).

Thus, following senescence induction, a small subpopulation of persistent cells emerged as a heterogeneous population composed of dividing and arrested clones.

### Secreted proteins increase senescence escape and transformation

**Fig. 2:**
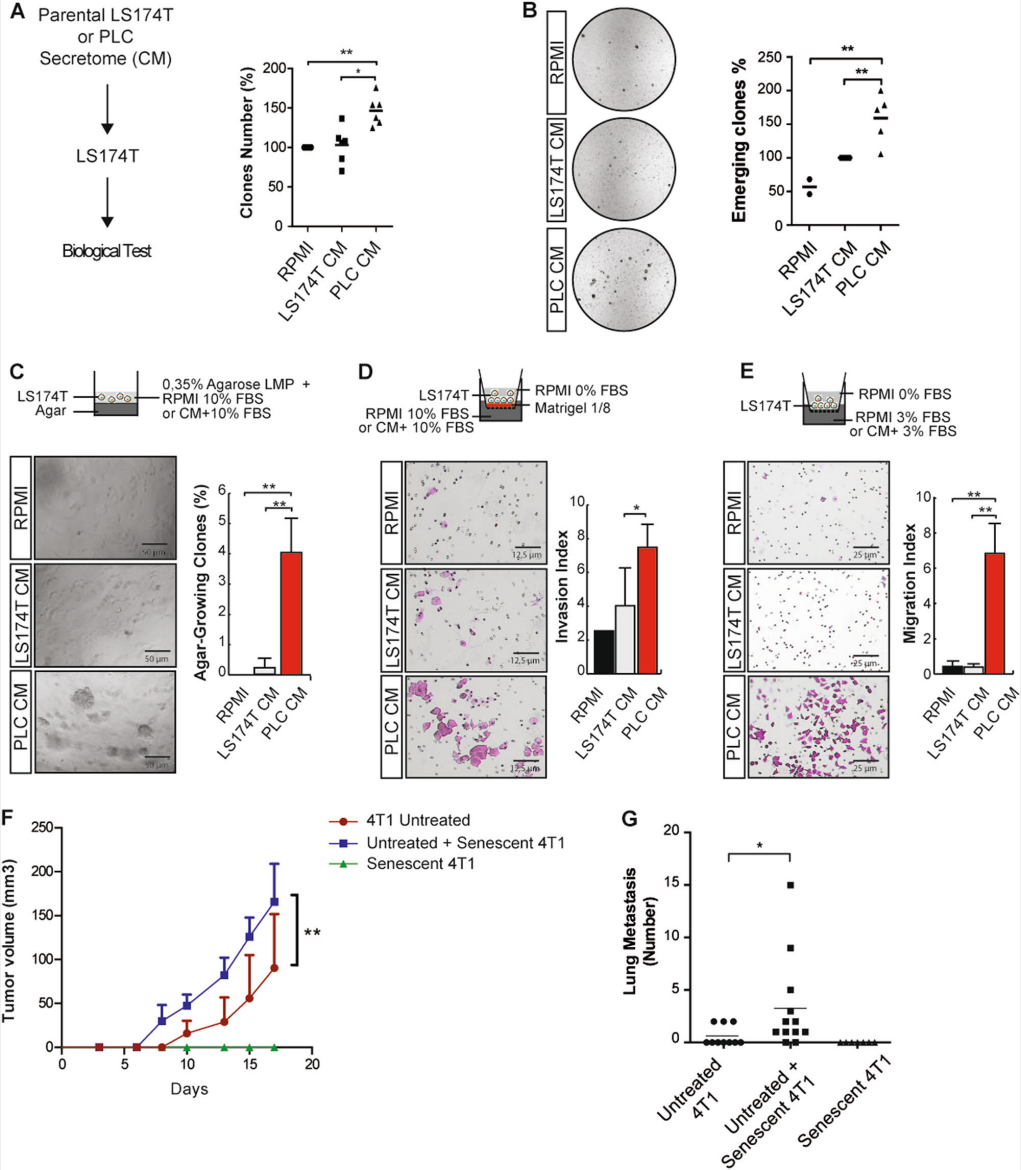
Emergence and cell invasion rely on secreted proteins produced by emergent cells. **a** Emergent cells were generated as described above and conditioned media (CM) collected after 24 h of serum starvation were supplemented with 10% FBS and applied to untreated parental LS174T cells. The proliferative capacity and survival were quantified by clonogenic tests (*n* = 6, Kolmogorov–Smirnov test, * = *p* < 0.05, ** = *p* < 0.01). **b** Conditioned media (CM) were collected after 24 h of serum starvation. LS74T cells were treated with sn38 for 4 days and emergence was induced after senescence induction for 7 days using either RPMI or conditioned medium from parental or emergent cells, both supplemented with 10% FBS. CM were added during the treatment and during emergence. Clones were then counted using crystal violet staining (*n* = 5, normalized to the emergence obtained from the CM of parental cells, Kolmogorov–Smirnov test, ** = *p* < 0.01). **c** Analysis of anoikis resistance and enhanced cell transformation using soft agar assays. Conditioned media obtained from parental or emergent cells were supplemented with 20% FBS and mixed with RPMI 0.7% low-melting-point (LMP) agarose to a final concentration of 10% FBS and 0.35% LMP agarose (*n* = 4). **d** Invasion assays were performed using Boyden inserts in which Matrigel was deposited at the bottom of the inserts. Conditioned media supplemented with 10% FBS were placed in the bottom chamber. After 72 h, invasive cells were stained with crystal violet (*n* = 5). **e** Migration assays were performed using Boyden inserts. Conditioned media obtained from parental or emergent cells supplemented with 3% FBS were placed at the well bottom. After 72 h, migrating cells were stained with crystal violet (*n* = 4). **f** CIS was induced in 4T1 cells following doxorubicin treatment (75 ng/ml, 4 days). Senescent cells mixed with untreated cells were injected in mice and tumor growth was monitored in the mammary fat pad of Balb/c mice and compared with controls (six mice were used for untreated 4T1 cells, seven for the mix of 4T1 cells and senescent population, and six for the senescent clones, two-way Anova with a Bonferroni’s multiple comparison test: ** = *p* < 0.01, *p* = 0.0018). **g** Following CIS induction, 4T1 cells were injected in the tail vein, alone or with senescent cells, and metastasis invasion was monitored after 31 days (Mann–Whitney test, * = *p* < 0.05)

To determine if secreted factors were involved in senescence escape, we exposed parental LS174T cells to a conditioned medium (CM) obtained from PLC emergent clones or untreated cells (Fig. 2a). Using clonogenic assays, we observed that the secretome from PLC increased cell division and CIS escape (Fig. 2a, b). We then used growth in soft agar as an indicator of increased cell transformation. A significant increase in spheroid number was observed, as compared with control supernatants (Fig. 2c). This secretome also significantly enhanced cell migration and invasion (Fig. 2d, e), and a maximal migration was observed 2 days after serum release (supplementary Figure 3A/B). This effect was also observed in MCF7 cells (supplementary Figure 3C). To confirm that senescent cells enhance oncogenic activity, the growth of parental cells mixed with senescent populations was analyzed in vivo. The murine breast 4T1 cell line was used since it grows efficiently in the mammary fat pad. Doxorubicin-treated cells entered senescence, as shown by SA-β-galactosidase staining and p21waf1 expression (data not shown). Using orthotopic grafting, we injected 4T1 cells, 4T1 cells mixed with a senescent 4T1 population (1–1 ratio) or senescent cells alone. The presence of the arrested population significantly enhanced the growth of untreated 4T1 cells (Fig. 2f). Increased lung metastasis was also observed when the two populations were co-injected into the tail vein, as compared with 4T1 untreated cells (Fig. 2g, supplementary Figure 4).

We concluded from these results that senescent cells produce an oncogenic secretome and that these cells favor cell emergence, cell transformation, and metastasis.

### Thrombospondin-1 is overexpressed in the emergent population and suppresses proliferation

**Fig. 3:**
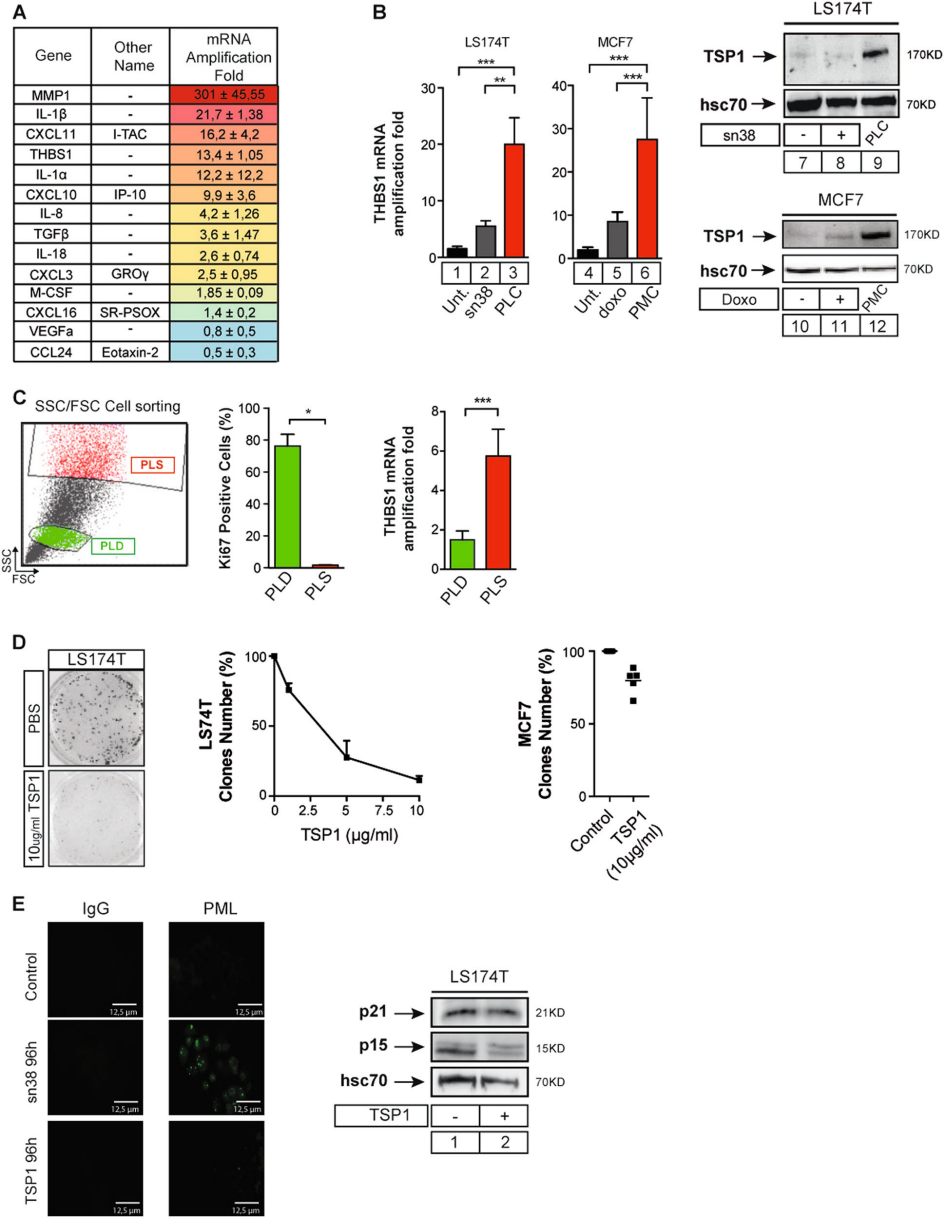
Thrombospondin-1 is overexpressed in senescent cells and blocks cell proliferation. **a** Quantitative RT-PCR analysis of SASP components in emergent cells (*n* = 3). Emergent cells were generated as described above and mRNA expression was analyzed as compared with parental cells. **b** Analysis of TSP1 expression, by RT-QPCR or by western blot in LS174T and MCF7 cells treated or not and after emergence. The presence of TSP1 in the secretome of emergent cells was assessed by western blot assay following serum starvation for 24 h. Intracellular hsc70 expression was evaluated in parallel on the corresponding adherent cells (*n* = 4, Mann–Whitney test, ** = *p* < 0.01, *** = *p* < 0.001). **c** Cell sorting of dividing PLD and senescent PLS clones according to FSC/SSC and Ki67 parameters (*n* = 4 +/− sd). The image illustrates the gates that have been used during cell sorting. THBS1 mRNA expression was analyzed by quantitative RT-PCR (*n* = 5 +/− sd, Mann–Whitney test, * = *p* < 0.05). **d** Analysis of LS174T (*n* = 3 +/− sd) and MCF7 (*n* = 5) proliferation and survival using clonogenic tests. Cells have been treated with the indicated concentrations of TSP1 or PBS for 7 days. **e** Following TSP1 stimulation of LS174T cells (10 µg/ml), PML staining and p21 and p15 expressions were evaluated by immunofluorescence and western blot (*n* = 3)

RT-QPCR experiments showed that classical SASP components^4,9^, such as IL1-alpha, IL1-beta, MMP, IL-8, and Gro were overexpressed in the emergent population (Fig. 3a). We also noticed that thrombospondin-1 was significantly upregulated. Since it has been recently shown that TSP1 mediates Ras-induced senescence^20^, we analyzed its function during CIS escape. The TSP1 mRNA was overexpressed in PLC (Fig. 3b, left), and western blot and ELISA experiments performed on supernatants showed that the protein was secreted from emergent colorectal and breast cells (Fig. 3b, Supplementary Figure 5). Using cell sorting, we can identify the PLD-dividing subpopulation within the emergent cells based on a low size and granularity profile and a high KI-67 staining. A high FSC/SSC profile was associated with KI-67 inhibition, p21waf1 upregulation, and SA-β-galactosidase staining and this identified the PLS senescent subpopulation (see refs. ^16,17^, Fig. 3c, supplementary Figure 3D). Using this approach, we observed that TSP1 was overexpressed in the PLS senescent subpopulation (Fig. 3c). Clonogenic tests indicated that this protein significantly reduced the proliferation of LS174T cells but less of MCF7 cells (Fig. 3d). TSP1 concentrations were chosen according to its level in the serum of patients (supplementary Figure 6B). We were not able to detect any PML bodies or upregulation of p21 or p15 (Fig. 3e, p16 is not expressed in LS174T or MCF7 cells), indicating that TSP1 did not induce senescence.

We therefore concluded that thrombospondin-1 is upregulated during senescence escape and that it inhibits cell proliferation.

### TSP1 prevents senescence escape and a low serum level is associated with tumor relapse

**Fig. 4:**
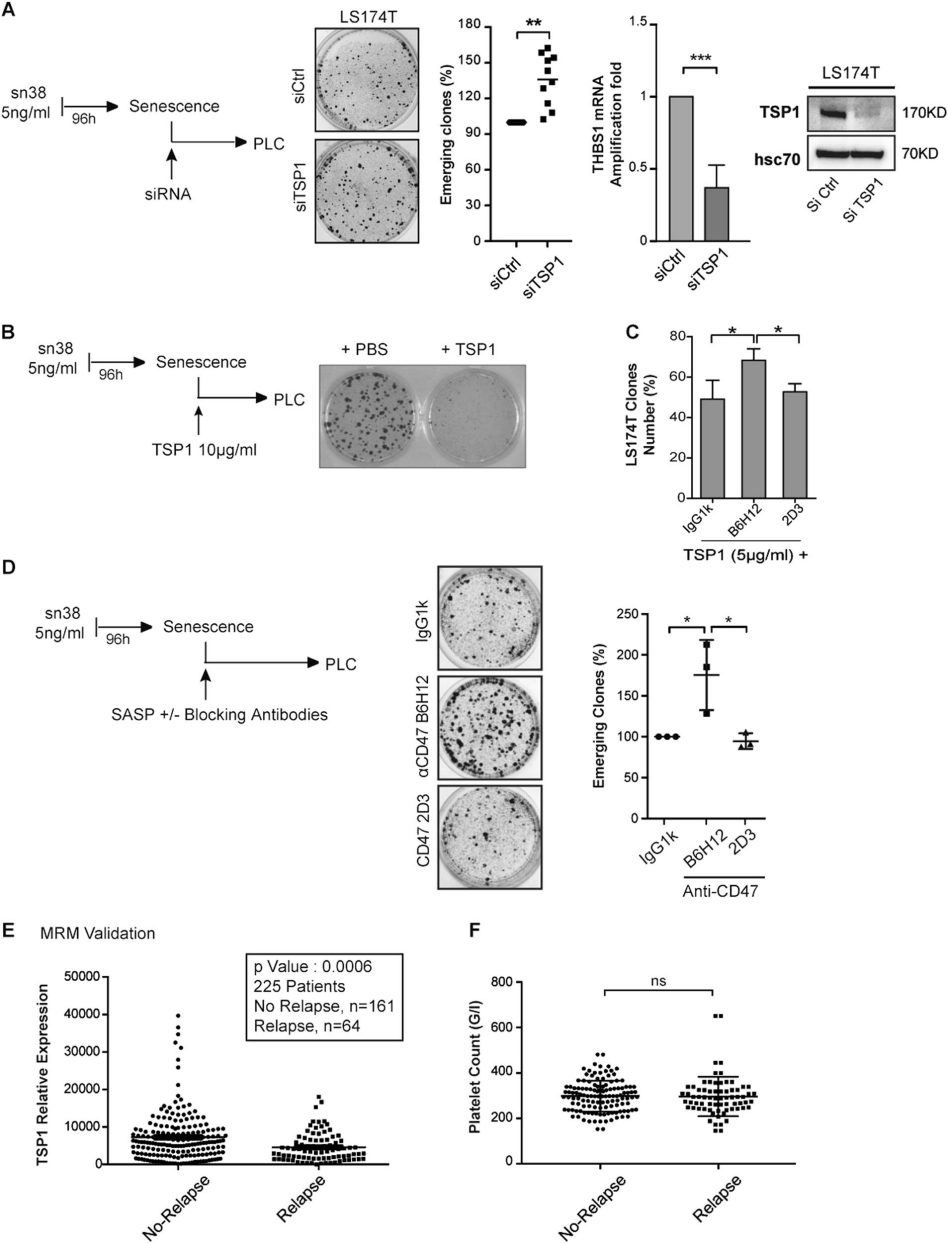
TSP1 prevents senescence escape. **a** After CIS induction, cells were transfected with siRNAs targeting TSP1. Transfection efficacy was assessed by evaluating THBS1 mRNA expression using quantitative RT-PCR or western blot analysis. Following a 24-h incubation, 10% FBS was added to induce emergence (Kolmogorov–Smirnov test, ** = *p* < 0.01, *** = *p* < 0.001). **b** After CIS induction, LS174T cells emergence was induced using 10% serum in the presence or absence of TSP1 (10 µg/ml, one representative image out of three experiments). **c** Cells were incubated for 5 mn with the indicated antibodies (B6H12 blocking antibody that prevents CD47–TSP1 binding, or its non-blocking control 2D3 or IgG1k isotype, all at 10 µg/ml). TSP1 (5 μg/ml) was then added to LS174T cells and clonogenic assays were performed for 7 days before crystal violet staining (*n* = 4 +/− sd, Kolmogorov–Smirnov test, * = *p* < 0.05). **d** Following treatment, emergence was induced using the secretome from PLC cells supplemented with 10% FBS and 10 µg/ml antibodies directed against CD47 or the control IgG1k isotype (*n* = 3 +/− sd, Kolmogorov–Smirnov test, * = *p* < 0.05). **e** MRM analysis and validation of TSP1 levels in patients that relapsed or not following chemotherapy treatment. Samples were obtained before chemotherapy treatment, see supplementary Figure 4 for the description of the clinical trial. **f** Analysis of platelet count in patients that relapsed or not following chemotherapy treatment

To determine if TSP1 regulates senescence escape, we then used siRNA to downregulate its expression, which was verified by RT-QPCR and western blot analysis (Fig. 4a). TSP1 downregulation significantly enhanced cell emergence (Fig. 4a). In addition, when added at the time of serum release, TSP1 inhibited CIS escape (Fig. 4b). TSP1 is known to interact with several receptors and we focused on the CD47 protein since this protein is involved in the response to irradiation^21^. Two different antibodies targeting CD47 were tested: the B6H12 antibody binds to the IgV domain of CD47 and prevents TSP1 binding, whereas the 2D3 clone binds to the transmembrane domain and does not prevent thrombospondin-1 signaling. Clonogenic tests showed that B6H12 significantly reduced the inhibitory effect of TSP1 on cell proliferation, whereas this was not the case with the 2D3 clone or the IgG1 control antibodies (Fig. 4c). We noticed however that the restoration of cell proliferation was not complete, indicating that other receptors might be involved. We then determined if the same effect could be observed during cell emergence. After senescence induction, escape was promoted using the secretome of emergent cells, in the presence or absence of the antibodies. The results show that the B6H12 antibody significantly enhanced cell emergence, but that the 2D3 control clone had no effect (Fig. 4d).

Since TSP1 reduces emergence, we then asked if this protein has any predictive value in the context of neoadjuvant chemotherapy. To test this hypothesis, we used blood samples collected from the PACS08 trial which included 225 patients diagnosed with poor prognosis triple-negative breast cancer (Trial ID: NCT00630032). The goal of this Phase III study was to compare three cycles of FEC100 (fluorouracil, epirubicin, and cyclophosphamide) followed by three cycles of docetaxel, with three cycles of FEC100 followed by three cycles of ixabepilone. Blood samples were obtained at inclusion, before any chemotherapy treatment (Supplementary Figure 6A). Owing to serum complexity, we used a new sensitive proteomic analysis (SWATH) to identify and quantify TSP1. By this approach, six TSP1 peptides were detected. All of these had a negative fold-change ratio Relapse versus No Relapse with a *p* value < 0.05 (log2 fold change = 1.22; *p* value = 0.01). This indicates that TSP1 was under-expressed in the serum of patients that relapsed from the disease. This result was then confirmed by the MRM approach (*p* value = 0.0006, Fig. 4e). On a limited number of patients, ELISA analysis indicated that the concentration of TSP1 in the serum varies between 10 and 30 µg/ml (Supplementary Figure 6B). Since TSP1 can be derived from platelets, we also determined if this correlation could be related to differences in platelet count. No significant differences were observed between no-relapse and relapse patients (Fig. 4f). In addition, we did not find any predictive value of TSP1 on survival (Supplementary Figure 6C)

Altogether, these results indicate that soluble TSP1 reduces senescence escape in vitro. In triple-negative breast cancer, chemotherapy failure was associated with a downregulation of its serum level.

### CD47^low^ cells have a reduced expression of p21waf1

**Fig. 5:**
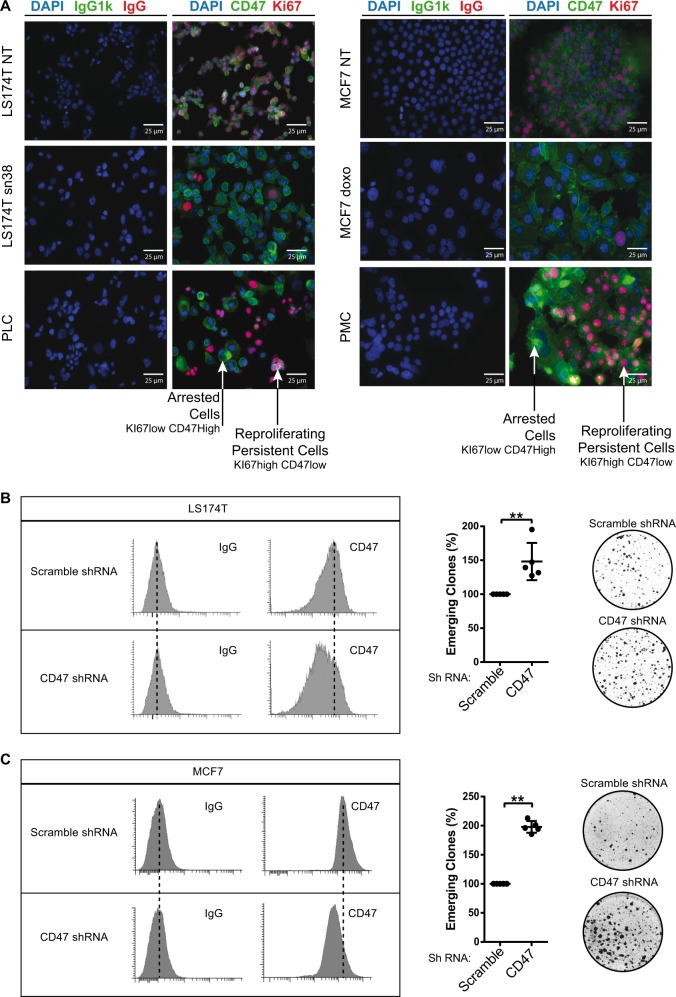
CD47 expression is downregulated in emergent clones. **a** Analysis of CD47 and Ki67 expressions by immunofluorescence in parental LS174T and MCF7 cells or emergent populations (PLC/PMC). Ki67 staining identifies the clones that have restarted proliferation in the middle of senescent cells (*n* = 3). **b** CD47 expression was analyzed by flow cytometry (one experiment representative of three, left part) in LS174T cells that express an shRNA directed against CD47 or a non-targeting control. In parallel, cells have been treated with sn38 to induce senescence and 10% FBS was added after 4 days to allow emergence (*n* = 3, Kolmogorov–Smirnov test, ** = *p* < 0.01). **c** CD47 expression was analyzed by flow cytometry in MCF7 clones expressing an shRNA directed against CD47 or a non-targeting control shRNA. Emergence was induced as described above (*n* = 3, Kolmogorov–Smirnov test, ** = *p* < 0.01)

We then determined if cell emergence was explained by a modulation of CD47 and TSP1. Using immunofluorescence staining, we observed that the PLD-dividing subclones (identified by KI-67 expression) expressed lower CD47 levels, as compared with the senescent PLS subpopulation (Fig. 5a). We also noticed that TSP1 was mainly expressed by senescent cells, whereas its expression was almost not detectable in dividing clones expressing Ki67 (Supplementary Figure 7A). To determine if CIS escape was explained by CD47 downregulation, cells were infected with an expression vector encoding a short-hairpin RNA targeting the receptor. CD47 downregulation was verified by FACS analysis (Fig. 5b, c). A significant increase of CIS escape was observed when CD47 expression was reduced, both in LS174T and MCF7 cells (Fig. 5b, c). This effect appears to be specific of senescence escape since this downregulation did not increase the proliferation of non-treated cells. Interestingly, the expression of the TSP1 mRNA was decreased following CD47 downregulation in senescent cells (Supplementary Figure 7B and C).

**Fig. 6:**
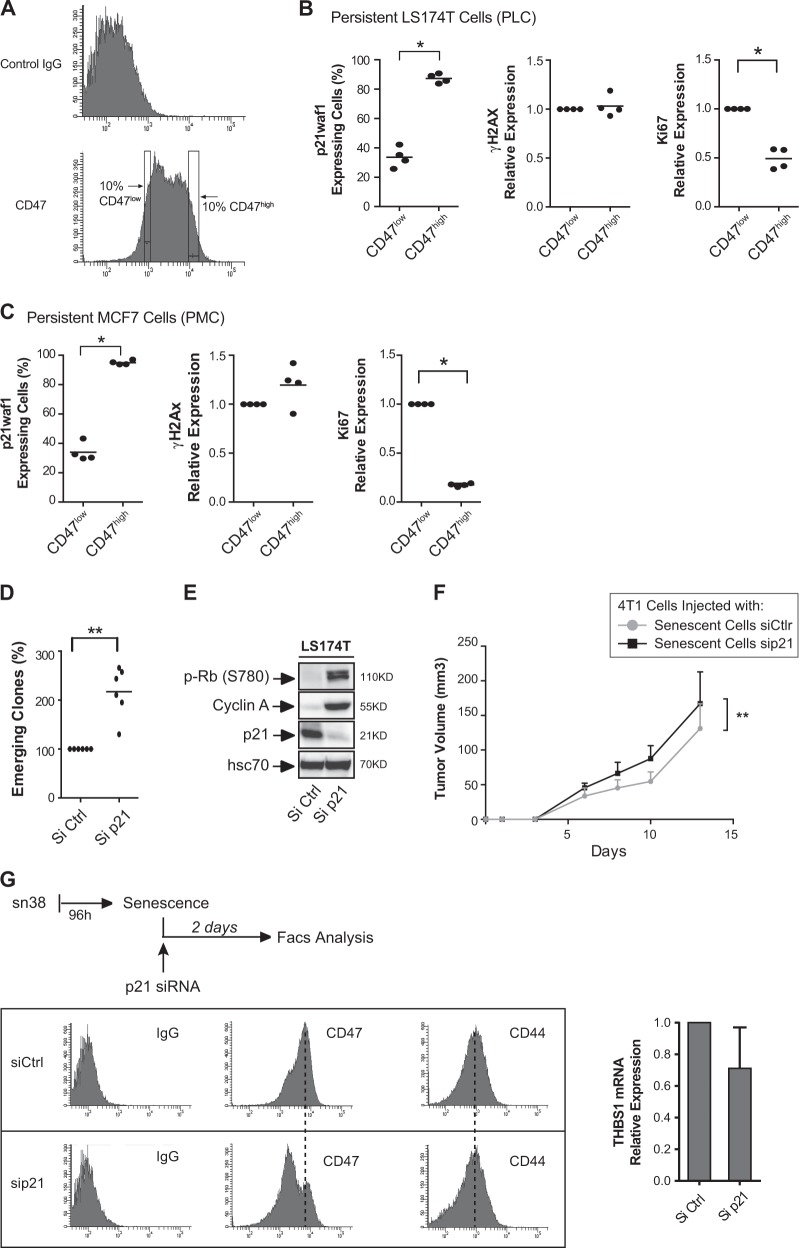
p21waf1 inactivation increases emergence and generates CD47^low^ cells. **a** Flow cytometry analysis of CD47 expression. The image describes the gating position of the 10% cells corresponding to CD47^low^ or CD47^high^ cells. **b**, **c** Following senescence induction, emergence was induced by serum addition. The dividing and senescent clones were then recovered and analyzed by intracellular flow cytometry. p21waf1, KI67, and ɣ-H2AX expressions were quantified by flow cytometry in CD47^low^ cells or CD47^high^ cells and normalized to control IgG staining (*n* = 4, LS174T (**b**) and MCF7 (**c**), Kolmogorov–Smirnov test, * = *p* < 0.05). **d** Senescence was induced as decribed above in LS174T cells and after 4 days, cells were transfected with control siRNA or siRNA directed against p21waf1. Ten-percent FBS was added to allow cell emergence (*n* = 6, Kolmogorov–Smirnov test, ** = *p* < 0.01). **e** LS174T cells were treated as above and cell extracts were recovered 2 days after p21waf1 inactivation by siRNA. The expression of the indicated proteins was analyzed by western blot (*n* = 4). **f** CIS was induced in 4T1 cells with doxorubicine and after 4 days, cells were transfected with a control siRNA or siRNA directed against p21waf1. After 24 h, cells were mixed with untreated 4T1 cells and injected in Balb/c mice (*n* = 7, two-way Anova with a Bonferroni’s multiple comparison test: ** = *p* < 0.01, *p* = 0.0075). Tumor growth was monitored in the mammary fat pad. **g** LS174T cells were treated as above to induce senescence and after 4 days, cells were transfected with control siRNA or siRNA directed against p21waf1. CD47 expression was analyzed by flow cytometry after 2 days and CD44 staining was used as a control (*n* = 4). In parallel, the expression of the THBS1 mRNA was analyzed by RT-QPCR (*n* = 3 +/− sd)

We then used intracellular flow cytometry to quantify senescence signaling in CD47^high^ and CD47^low^ emergent cells. These populations were defined, respectively, as the 10% of emergent cells expressing the highest or the lowest level of the receptor (Fig. 6a). Flow cytometry results indicate that CD47^low^ cells showed a decreased expression of p21waf1 and a higher level of KI67, as compared with CD47^high^ cells (Fig. 6b). No difference in ɣ-H2Ax staining was noticed between these two subpopulations, either during emergence or in senescent cells (data not shown). This indicates that CIS escape of the CD47^low^ subpopulation is not due to reduced DNA damage. The same observation was made using MCF7 cells. Emergent CD47^low^ breast cells also showed a reduced p21waf1 expression and a higher level of KI67 (Fig. 6c).

Taken together, these results indicate that CD47^low^ cells escape senescence with a reduced expression of p21waf1 and a higher expression of KI67.

### p21waf1 maintains senescence and prevents the generation of CD47^low^ cells

We then determined if p21waf1 was necessary to maintain senescence. To this end, the cell cycle inhibitor was downregulated by RNA interference following senescence induction. This led to a significant increase in cell emergence (Fig. 6d), inducing Rb phosphorylation and cyclin A upregulation (Fig. 6e). Note that we have previously described that the expression of the cell cycle inhibitor gradually decreased in persistent cells^17^. To extend these results, we induced senescence in 4T1 cells, then we downregulated the cell cycle inhibitor by siRNA, and injected this population in vivo with untreated 4T1 cells. p21waf1 inactivation significantly enhanced the oncogenic effect of senescent cells (Fig. 6f). Interestingly, p21waf1 inactivation in LS174T senescent cells increased the proportion of the CD47^low^ subpopulation (Fig. 6g). No effect was observed on CD44 expression, but the results showed a slight reduction of the THBS1 mRNA expression. The same effect was observed in senescent MCF7 cells where p21waf1 inactivation also increased CIS escape and led to CD47 downregulation (supplementary Figure 8A–C). This effect was specific of senescence escape, and p21waf1 downregulation did not increase the proliferation of non-treated cells and did not modify the expression of CD47 in this condition (Supplementary Figure 8D/E).

Overall, these results indicate that p21waf1 is necessary to maintain senescence and that its downregulation generates CD47^low^ cells and induces cell emergence.

### p21waf1 prevents the generation of CD47^low^ cells by downregulating Myc expression

**Fig. 7:**
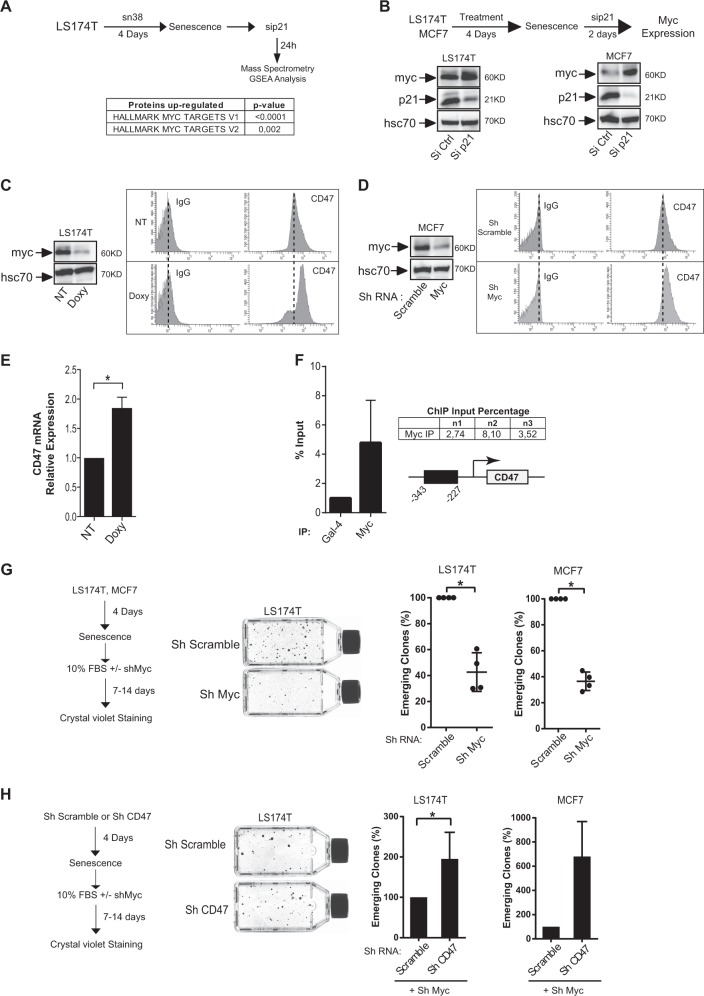
p21waf1 prevents the generation of CD47^low^ cells by downregulating Myc expression. **a** Following senescence induction, emergence was induced by serum addition, in the presence or absence of a control siRNA or a siRNA directed against p21 (*n* = 3). Cell extracts were analyzed by SWATH quantitative proteomics and GSEA analysis. **b** Validation of Myc induction by western blot analysis following p21 downregulation and emergence (*n* = 3). **c**, **d** Myc expression was downregulated by an inducible shRNA in LS174T cells or by a transient infection of a different shRNA in MCF7 cells. CD47 expression was then analyzed by flow cytometry. One experiment representative of 4 is presented. **e** Myc was downregulated as described above in LS174T cells and the expression of the CD47 mRNA was evaluated by RT-QPCR (*n* = 4 +/− sd, Kolmogorov–Smirnov test, * = *p* < 0.05). **f** ChIP assays were performed following Myc or Gal4 immunoprecipitation using the indicated primers and analyzed by quantitative PCR (*n* = 3). Each amplification is also shown compared with the Gal4 signal. **g** Senescence was induced as decribed above and after 4 days, 10% FBS was added to allow LS174T or MCF7 cell emergence, in the presence of control shRNAs or shRNAs directed against Myc (*n* = 4, Kolmogorov–Smirnov test, * = *p* < 0.05). **h** LS174T or MCF7 cells were infected with a lentivirus expressing an shRNA targeting CD47 or a control sequence. Senescence was then induced as described and after 4 days, 10% FBS was added to allow LS174T or MCF7 cell emergence, in the presence or absence of control shRNA or shRNA directed against Myc (LS174T (*n* = 4), MCF7 (*n* = 3), Kolmogorov–Smirnov test, *p < 0.05)

Using quantitative proteomic and GSEA analysis, we detected a significant activation of Myc signaling in emergent cells following p21waf1 inactivation (Fig. 7a). This upregulation was validated by western blot (Fig. 7b), confirming previous results by our group and others which described p21waf1 as a repressor of proliferative genes^22,23^. This result suggested that Myc reactivation during emergence might generate CD47^low^ cells. To test this hypothesis, we inhibited the transcription factor by two approaches, using an inducible shRNA in LS174T cells^24^ or a transient and different shRNA infection in MCF7 cells. FACS experiments indicated that Myc downregulation led to an increase in CD47 expression (Fig. 7c, d). This effect occured at the transcriptional level since its mRNA was also increased (Fig. 7e). Chromatin immunoprecipitation experiments showed that Myc was recruited to the proximal CD47 promoter (Fig. 7f). In addition, its downregulation significantly reduced the number of emergent cells following senescence induction (Fig. 7g). As expected, Myc also reduced the proliferation of non-treated cells, which indicate that its effect on cell emergence is probably also related to its well-known control of cell division (Supplementary Figure 9A). Interestingly, RT-QPCR experiments also showed that Myc downregulation led to an upregulation of the TSP1 mRNA (Supplementary Figure 9B).

We then determined if the upregulation of CD47 was important in myc-mediated effect on senescence escape. To this end, we first downregulated CD47 expression and further infected cells with a lentivirus expressing an shRNA directed against Myc. Myc downregulation increases the endogenous CD47 expression to produce Myc^low^CD47^high^ cells. This should be reduced by the CD47shRNA to generate Myc^low^CD47^low^ cells. If the repression of the receptor is indeed important for emergence, Myc^low^CD47^low^ cells should escape senescence despite Myc downregulation. Effectively, the results obtained both in LS174T colorectal and mammary MCF7 cells indicate that CD47 downregulation effectively restored cell emergence, despite Myc inactivation (Fig. 7h). This suggests that the effect of Myc on emergence is not only mediated by its function on cell division, but that its transcriptional repression of CD47 is also involved in senescence escape.

Overall, these results indicate that p21waf1 maintains senescence by preventing Myc upregulation and the consequent generation of CD47^low^ cells.

### Primary cells escape senescence with a reduced expression of CD47 and p21waf1

**Fig. 8:**
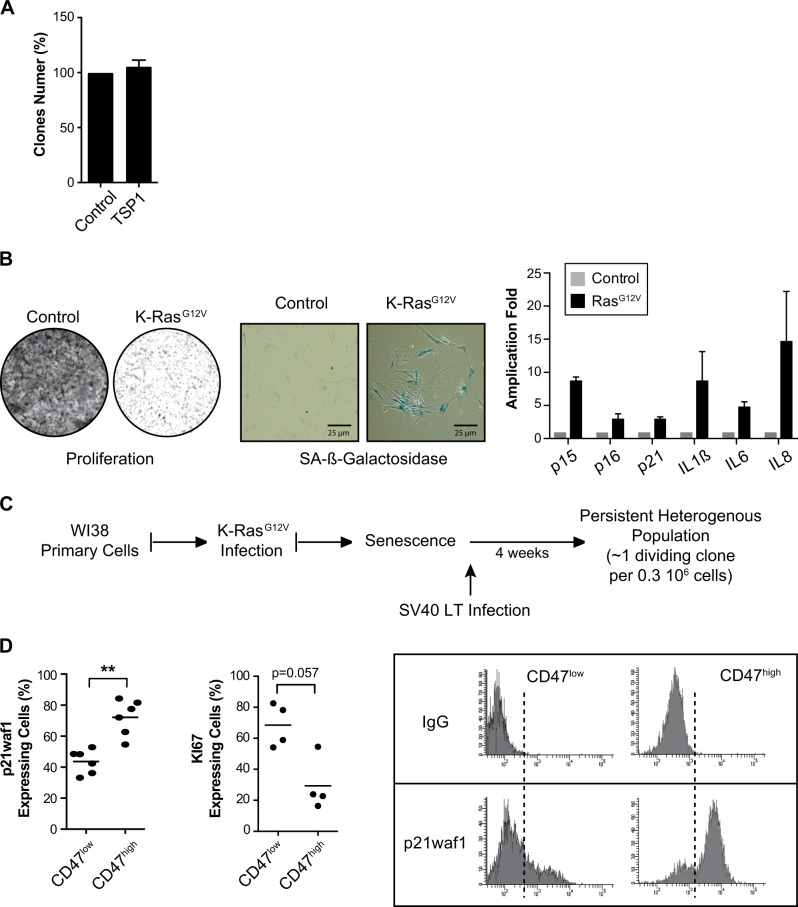
Senescence escape in primary fibroblasts generates CD47^low^ cells with a reduced expression of p21waf1. **a** Analysis of WI38 proliferation and survival using clonogenic tests (*n* = 3 +/− sd). Cells have been treated with TSP1 (10 µg/ml) or PBS for 7 days. **b** WI38 cells have been infected with a lentivirus expressing a K-Ras^G12V^*-*estrogen receptor chimera. After KRas^G12V^ induction, senescence was evaluated at day 20 by crystal violet staining (left), SA-β-galactosidase activity (middle), and mRNA expression of p15INK4b, p16INK4a, p21waf1, IL1-beta, IL-6, and IL-8 (*n* = 5 +/− sd). **c** Senescence was induced in WI38 cells as described above and cells were then infected with a lentivirus expressing the SV40 large T antigen or GFP as a control. Emergence was observed after 1 month. **d** One week after emergence detection, dividing and senescent WI38 clones were recovered and analyzed by intracellular flow cytometry. p21waf1 and KI-67 expressions were quantified by flow cytometry in CD47^low^ cells or CD47^high^ cells and normalized to control IgG staining (*n* = 6, *n* = 4 for KI-67 staining, Kolmogorov–Smirnov test, ** = *p* < 0.01). One illustrative image of p21waf1 staining is presented

We confirmed these results in primary WI38 pulmonary fibroblasts. Using clonogenic tests, we did not observe any effect of TSP1 on proliferation (Fig. 8a). To determine if CD47^low^ cells were involved in senescence escape, WI38 cells were infected with a lentivirus expressing a K-Ras^G12V^*-*estrogen receptor chimera. As expected, 4-hydroxytamoxifen (4-HT) induction led to growth arrest, SA-β-galactosidase staining, upregulation of p15INK4b, p16INK4a, and p21waf1, and induction of IL-6 and IL-8 (Fig. 8b). To induce senescence escape as previously described^25^, we infected arrested cells with the SV40 large T antigen (Fig. 8c). After 1 month, persistent clones restarted proliferation (around one or two clones per 300,000 cells). As described above for LS174T and MCF7 cells, escape led to the emergence of a heterogeneous population composed of dividing and arrested cells. Following senescence escape, we used intracellular flow cytometry to quantify KI67 and p21waf1 expression in CD47^low^ and CD47^high^ emergent cells. The results indicate that CD47^low^ cells also showed a decreased expression of p21waf1 and a higher level of KI67, as compared with CD47^high^ cells (Fig. 8d).

Taken together, these results indicate that primary cells also escape from Ras-mediated senescence by generating CD47^low^ cells with a reduced expression of p21waf1 and a higher expression of KI67.

## Discussion

By definition, tumor-suppressive mechanisms have to be inactivated during the successive steps of cell transformation. For this reason, in the late stages of the disease, CIS is probably not a complete suppression but more of an adaptive mechanism that could favor the emergence of cell subpopulations. Indeed, we have shown that colorectal or mammary cells can escape CIS and emerge as more aggressive populations^14–18^. It will be interesting to determine if this escape can be extended to other cancer cells. In particular, we have observed that p21waf1 upregulation during the acute response to DNA damage is essential to allow senescence escape^16^. Further experiments are therefore necessary to determine if senescence is indeed a general adaptive pathway to chemotherapy, if this concerns only a specific subset of cancer cells and how this relies on the integrity of p53, p21, and p16 signaling or on specific oncogenic pathways. Our results show that CIS escape is mediated by secreted proteins and that emergent cells favor tumor cell growth and invasion. Since emergence produces a heterogeneous population, we propose that the senescent population provides a surviving niche for dividing clones. Senescent cells communicate with neighboring clones through the SASP but the outcome of this secretome varies considerably. We identified TSP1 as a new secreted factor upregulated in senescent cells that prevents cell emergence. This study therefore uncovers a new function of this protein during senescence escape, in experimental models but also in vivo since chemotherapy failure was associated with the downregulation of its serum level. We propose that TSP1 detection in serum will be useful to predict tumor relapse. Further experiments are now necessary to confirm this predictive result on a larger cohort and to determine if TSP1 detection can be also useful to predict the occurrence of irinotecan-refractory colorectal cancer.

Our results also raise the question of the function of CD47 in the context of tumor relapse. It is important to note that TSP1 binds to several proteins and that one should not conclude from this study that these effects are only mediated by the CD47–TSP1 interaction. Preventing this binding restores cell proliferation and increases SASP-mediated emergence, but other TSP1 targets are certainly also involved in the control of senescence escape. Our results describe the generation of an emergent population expressing p21waf1 to a lower extent than arrested cells. The downregulation of the cell cycle inhibitor leads to Myc upregulation which then represses CD47 to generate a CD47^low^ subpopulation that escape senescence (supplementary Figure 10). It has already been reported that CD47 is involved in chemotherapy response. For example, its inactivation significantly potentiates the effect of radiation on the growth of melanoma cells^21^. It has been shown that CD47 is expressed in cancer-initiating cells and that this receptor is involved in metastasis progression^26,27^. In addition, targeting CD47 inhibits the proliferation of breast CD44^high^CD24^low^ cancer stem cells^28^.

CD47 overexpression has been reported in most tumor types, including leukemia, lymphoma, and solid tumors. This protein binds to the signal regulatory protein alpha (SIRPα) expressed on macrophages and prevents the elimination of tumor cells. Preventing the binding between SIRPα and CD47 by B6H12 antibodies enhances cancer cell killing by immune cells. As a result, several CD47 antibodies are used in clinical trials to enhance antitumor immunity. In light of the unexpected role of the CD47 pathway in CIS control, we propose that targeting CD47 in combination with chemotherapy should be tested with caution. Since our results and a previous study indicate that this pathway is activated in triple-negative breast cancer^28,29^, further experiments are necessary to determine the benefit of targeting CD47 in specific cancer subtypes that may respond differently to genotoxic treatments, depending on the level of TSP1 and of senescence induction.

## Methods

### Cell lines, senescence induction, and generation of persistent cells

All cells were obtained from the American Type Culture Collection. The human recombinant thrombospondin-1 (rhTSP1) was obtained from R&D Systems (3074-TH-050) and reconstituted at 100 µg/ml with PBS before use. Cell lines were authenticated by STR profiling and were regularly tested to exclude mycoplasma contamination. To induce senescence, all cells were treated for 96 h with sn38 (5 ng/ml, LS174T) or doxorubicin (25 ng/ml for MCF7, 75 ng/ml for 4T1). In WI38 cells, senescence was induced following KRas^G12V^ induction for 96 h. Senescence was validated by expression of cell cycle inhibitors, β-galactosidase staining, DNA damage, and inhibition of proliferation. For generation of persistent cells, LS174T, MCF7, and 4T1 cells were treated for 4 days with sn38 (5 ng/ml) and doxorubicin (25 ng/ml for MCF7, 50 ng/ml for 4T1) in 3% FBS, washed with PBS, and then restimulated with fresh 10% FBS for 7 days. WI38 cells were infected with a lentivirus expressing SV40 large T antigen, following senescence induction by KRas^G12V^. The next day, the media was replaced with fresh 10% FBS, and clones took around 3/4 weeks to emerge. To use supernatants, cells were serum-starved for 24 h with fresh serum-free media. Conditioned media (CM) were collected and centrifuged twice at 200 × *g* for 5 min to eliminate cell debris. WI38 primary cells were used between passage 22 and 30.

### Treatment

Colorectal cancer cells were treated with sn38 (Tocris Bioscience 2684), the active metabolite of irinotecan which is a hemisynthetic analog of camptothecin. Breast cancer cells were treated with doxorubicin (Tocris Bioscience 2252), a biosynthetic analog of daunorubicin. As topoisomerase I and II inhibitors, respectively, these drugs prevent the DNA religation and are responsible for DNA single- or double-strand breaks. These DNA damages lead to senescence in our conditions.

### Preparation of blocking antibodies

Blocking antibodies were purchased from eBioscience: anti-CD47 clone B6H12 (14-0479-82), anti-CD47 clone 2D3 (14-0478-82), and control isotype IgG1k (14-4714-82). To remove sodium azide, antibodies were washed with PBS and filtered three times through centrifugal units for 5 min at 14,000 × *g* (Millipore, Amicon, UFC503096) previously sterilized with 70% ethanol. Concentration was then determined with the BCA protein assay kit (Kit Pierce™ BCA Protein Assay, Thermofisher). Cells were treated with 1–10 µg/ml of blocking antibodies.

### siRNA inactivation

Cells were transfected with 50 nM small interfering RNA against thrombospondin-1 (Santa Cruz, sc-36665), p21waf1 (ON-TARGET plus Human CDKN1A (1026), Dharmacon, L-00341-00-0005), mouse Cdkn1a siRNA: Dharmacon, ref L-058636-00-0005, and prevalidated control siRNA (Dharmacon, D-001810-10-20) using DharmaFect-4 (Dharmacon).

### Invasion and migration assay

Cells were harvested and resuspended in serum-free culture medium. In the invasion assay, a thin (~1-mm) layer of Matrigel (ECM gel, Sigma) was coated at the bottom of the insert pierced with a 8-µm pore size (Falcon). In this case, Matrigel was diluted to 1/8 with serum-free medium. In both experiments, 200,000 cells/ml of cell suspension was prepared, of which 200 µl were deposited in each insert. Below the inserts, 800 µl of 3% (migration assay) or 10% (invasion assay) FBS medium (RPMI or CM) were placed and cells were incubated for 72 h. Then, cells were fixed, washed with PBS, and stained with crystal violet. Cells in the inner compartment were removed and cells on the outside were counted with ImageJ software.

### Animal studies

They were conducted in strict accordance with the principles and procedures approved by the local Ethics Committee. *Xenografts in NOD-SCID mice*: female NOD-SCID mice were inoculated subcutaneously in the right flank with 1×10^6^ parental, senescent, LS174T, or PLC cells. The tumor volume was monitored every 2–3 days until killing. An electronic caliper was used to measure the length (*L*) and width (*W*) of the tumors. Tumor volume was estimated by applying the following equation: (3.14/6) × *L* × *W*^2^. *Orthotopic allografts in Balbc mice:* 8-week-old female Balbc mice were inoculated in the right secondary fat pad with 1×10^4^ parental cells, mixed or not with 1×10^4^ senescent cells, or with 2 ×10^4^ senescent cells alone. The monitoring and measuring was carried out as described above. *Metastatic study:* 8-week-old female Balbc mice were inoculated in the caudal vein with 1×10^4^ parental cells mixed or not with 1×10^4^ senescent cells, or with 2 × 10^4^ senescent cells alone. One month later, lungs were recovered and analyzed for metastatic colonization by a pathologist.

### Mass spectrometry analysis

See supplementary text.

### Immunofluorescence

#### PML staining

Cells were fixed with 2% formaldehyde for 10 min at room temperature and then permeabilized by a 30-min incubation in cold 70% ethanol at 4 °C. After washing three times in PBS–Tween 0.02% and blocking in PBS–BSA 2% during 15 min, the cells were incubated for 4 h at room temperature with an anti-PML antibody (mouse monoclonal IgG_1_, Santa Cruz: sc-966 (1/50)) diluted in the blocking buffer. After washing three times in PBS–Tween 0.02% and blocking in PBS–BSA 2% during 15 min, cells were incubated with the secondary antibody diluted 1/200 (goat anti-mouse IgG secondary antibody Alexa 488, Invitrogen-Molecular Probes: A11001) for 1 h at room temperature in the dark. Cells were then washed three times in PBS–Tween 0.02%, and covered with the anti-fading reagent with DAPI (ProLong Diamond Antifade Mountant with DAPI, P36962).

#### Double staining for CD47, TSP1, and Ki67

After fixation with 2% formaldehyde for 10 min, permeabilization with 70% cold ethanol, and a blocking step, cells were incubated simultaneously for 4 h with the following antibodies diluted 1/100: mouse IgG1k isotype control (eBioscience, 14-4714-82), XP rabbit mAb isotype control IgG (Cell Signaling, 3900S), anti-CD47 B6H12 IgG1k mouse (eBioscience, 14-0479), thrombospondin-1 (Cell Signaling, 37879), and anti-Ki67 IgG rabbit (Cell Signaling, 9129S). After washing three times with PBS–Tween 0.02%, cells were incubated with secondary antibodies diluted 1/200: goat anti-mouse IgG secondary antibody Alexa 488 (Invitrogen, A11001) and goat anti-rabbit IgG secondary antibody Alexa 568 (Invitrogen, A11011). Cells were washed three times with PBS–Tween 0.02% and covered with Prolong Diamond with DAPI.

### Statistical analysis

All values were expressed as mean +/− standard deviation (SD). Differences were analyzed using nonparametric tests (*t* test, Mann–Whitney, Kolmogorov–Smirnov, and one-way Anova). *p < 0.05, **p < 0.01, and ***p < 0.001. NS on the figure means that the result was not significant.

### Quantitative PCR

Analyses were performed with normalization of the target gene expression to the endogenous housekeeping genes RPLPO, G3PDH, beta-actin, and TBP. PCR primer sequences were as follows:

**Table:** 

Protein	Gene	Forward 5′–3′	Reverse 5′–3′
G3PDH	GAPDH	GAAGGTGAAGGTCGGAGTC	GAAGATGGTGATGGGATTTC
RPLP0	RPLP0	AACCCAGCTCTGGAGAAACT	CCCCTGGAGATTTTAGTGGT
Beta-actin	Beta-actin	TGGAGAAAATCTGGCACCACACC	GATGGGCACAGTGTGGGTGACCC
TBP	TBP	TGCACAGGAGCCAAGAGT GAA	CACATC ACA GCT CCC CAC CA
TSP1	THBS1	AGGGCAGGAAGACTATGACAAG	GCTGGGTTGTAATGGAATGG
P15	CDKN2B	GAATGCGCGAGGAGAACAAG	CCATCATCATGACCTGGATCG
P16	CDKN2A	CGGTCGGAGGCCGATCCAG	GCGCCGTGGAGCAGCAGCAGCT
P21	CDKN1A	GCTCCTTCCCATCGCTGTCA	TCACCCTGCCCAACCTTAGA
IL1-beta	IL-1B	GGCCCTAAACAGATGAAGTGCT	TGCCGCCATCCAGAGG
IL-6	IL-6	AAGATGTAGCCGCCCCACACA	CTGCCAGTGCCTCTTTGCTGCT
IL-8	IL-8	TTGGCAGCCTTCCTGATTTC	TCTTTAGCACTCCTTGGCAAAAC

### Western blotting

The following antibodies were used: anti-p21Waf1 (Cell Signaling, 2947S), anti-TSP1 (Santa Cruz, sc-12312), anti-p15 (Santa Cruz, sc-612), anti-p-Rb (S780) (BD Pharmingen, 558385), anti-cyclin A (Santa Cruz, sc-271282), rabbit polyclonal anti-Myc (Cell Signaling, 5605S), and anti-hsc-70 (Santa Cruz, sc-7298). Visualization was performed by chemiluminescence with a Bio-Rad Chemi Doc XRS imaging device (Bio-Rad) and Fusion Solo (Vilber).

### Soft agar assay

Note that cell preparation is a very sensitive step. LS174T cells were plated in 3% FBS for 4 days. Then, adherent cells were removed with trypsin and resuspended in 10% FBS culture medium. A cell suspension was prepared with 1×10^6^ cells/mL, in 100 μL of 10% FBS culture medium. In total, 2.5 or 5 μL of this, containing 2500 or 5000 cells, were then transferred to a 1.5-mL tube. These cells were suspended in 100 μl of a preheated mix containing 0.35% low-melting agarose (Promega), 10% FBS medium (fresh RPMI or conditioned medium), and antibiotics and then seeded on the lids of agar-coated 96-well plates. The base agar layer contained 50 μl of 1% agarose (Invitrogen) in 10% FBS culture medium with penicillin–streptomycin (Lonza). Ten days after incubation at 37 °C with 5% CO_2_, cell clones were counted and images were made. Each sample was tested in three technical replicates. Representative pictures were shown and the average of these three replicates was used for the clone count.

### Flow cytometry

Cells were trypsinized, washed with PBS, and 125,000 cells were incubated for 15 min with 200 ng of APC anti-CD47 (eBiosciences, 17-0479-42) or 200 ng of APC mouse IgG1K isotype control (eBiosciences, 17-4714-42) in the dark at room temperature. When only the membrane expression of the receptor was investigated, cells were washed and directly analyzed on the cytometer. To investigate the correlation between CD47 membrane expression and intracellular proteins, cells were then fixed and permeabilized.

#### Fixation and permeabilization

Cells were incubated with 4% paraformaldehyde for 10 min at 37 °C. Then, cells were washed and permeabilized with cold 90% methanol for 30 min in ice. Cells were washed and stained for intracellular proteins as described below. The staining solutions were prepared in PBS–BSA 2%.

#### P21 staining

Cells were incubated with 88 ng of rabbit IgG anti-p21 (Cell Signaling, 2947S) or 88 ng of rabbit IgG control isotype (Cell Signaling, 3900S) for 1 h in the dark at room temperature. Then, cells were washed and stained with 500 ng of the Alexa Fluor 488 (A488) goat anti-rabbit IgG (Invitrogen, A11008) for 1 h in the dark at room temperature.

#### p-H2AX (Ser 139) staining

Cells were incubated with 16 ng of A488 mouse anti-p-H2AX (Ser 139) (BD Pharmingen, 560445) or 16 ng of A488 mouse IgG1K (BD Pharmingen, 557721) for 1 h in the dark at room temperature.

#### CD44 staining

Cells were incubated with 15 ng of APC mouse anti-human CD44 (BD Pharmingen, 5599942) or 15 ng of APC mouse IgG2b K isotype control (BD Pharmingen, 555745) for 20 min at room temperature.

#### Ki67 staining

Cells were incubated for 30 min at room temperature in the dark with 5 µl of the antibody against Ki67 or isotype control of the FITC Mouse anti-Human Ki-67 Set (BD Pharmingen, 556026).

### shRNA, lentiviruses, and cell transduction

pLenti-PGK-ER-KRAS(G12V) was a gift from Daniel Haber (Addgene plasmid # 35635). Lenti-sh1368 knockdown c-myc was a gift from Bob Eisenman (Addgene plasmid # 29435). Lentiviruses were packaged in 293T cells by cotransfection with the packaging plasmid (pMDLg/pRRE, pRSV-Rev, and PMD2.G) and the different PLKO constructs. At 48 h post transfection, viral media was collected, centrifuged and filtered through 0.45 µm, and transduced into cells in the presence of 4 µg/ml of polybrene (Santa Cruz). Infected LS174T and MCF7 cells were incubated for 24 h and then given fresh growth media for 48 h. MCF7 cells were selected with 5 µg/ml puromycin (Sigma-Aldrich), and LS174T cells were used in the absence of selection.

**Table:** 

Vector	Target gene	Source	Sequence
pLKO.1	CD47	MISSION shRNA Sigma TRCN0000007839	CCGGGCACAATTACTTGGACTAGTTCTCGAGAACTAGTCCAAGTAATTGTGCTTTTT
pLKO	Nontarget shRNA control	MISSION shRNA Sigma SHC216	Nontarget shRNA control

### ELISA assay

TSP1 concentration was determined using ELISA kits from Fischer Scientific (ref 15561657).

### Clonogenic assay

Assays were performed to evaluate proliferation and survival. In total, 286 LS174T and 180 MCF7 cells were seeded into 24-well cell culture plates with 3% FBS, in the presence or absence of TSP1; note that TSP1 was added on floating cells, the first day. Cells were then incubated at 37 °C in a 5% CO_2_ atmosphere for 7 days, then washed twice with PBS, and stained with 0.4% crystal violet. The colonies were then washed twice with water, visualized with a Bio-Rad Chemi Doc XRS Imaging device, and counted using Quantity One imaging software (Bio-Rad).

### Chromatin immunoprecipitation (ChIP) assay

Cells were cross-linked with 1% formaldehyde for 10 min at room temperature. Cross-linking was stopped by adding 0.125 mol/L glycine for 5 min. Cells were washed three times with cold phosphate-buffered saline (PBS). Cells were then scraped and washed three more times with cold PBS. Pellets were resuspended in 1 mL of lysis buffer (5 mM PIPES, 85 mM KCl, and 0.5% NP40). All buffers were supplemented with proteases and phosphatase inhibitors (1 mM PMSF, 10 μg/ml aprotinin, 10 μg/ml leupeptin, 10 μg/ml pepstatin, 1 mM Na_3_VO_4_, and 50 mM NaF). Samples were incubated for 15 min at 4 °C and vortexed for 30 s every 2 min. Cells were centrifuged for 10 min, 12,000 rpm at 4 °C. Supernatants were discarded and pellets were resuspended in 500 µL of sonicating buffer (10 mM EDTA, 1% SDS, and 50 mM Tris-EDTA, pH = 8). Nuclei extracts were sonicated (four cycles of 25 s of sonication and 25 s in ice) to obtain DNA fragments of about 500–1000 pb. Supernatants were recovered by centrifugation at 12,000 rpm for 10 min at 4 °C and diluted 10 times with IP buffer (0.01% SDS, 1.1% Triton X-100, 1.2 mM EDTA, 16.7 mM Tris-HCl (pH = 8.0), and 167 mM NaCl).

For each 500-μL extract, 5 µL of DTT (0.1 M) and 15 μl of ChIP-grade Protein A/G Magnetic beads (ThermoFisher), beforehand conjugated with 2 μg of specific antibodies were added. After incubation overnight at 4 °C, the beads were washed with 600 µL of TSE1 buffer (1% Triton X-100, 150 mM NaCl, 20 mM Tris-HCl, pH 8.1, 0.1% SDS, and 2 mM EDTA), 600 µL of TSE2 buffer (1% Triton X-100, 500 mM NaCl, 20 mM Tris-HCl, pH 8.1, 0.1% SDS, and 2 mM EDTA), and 600 µL of TSE3 buffer (1% NP40, 1% sodium deoxycholate, 250 mM LiCl, and 10 mM Tris-HCl, pH 8.1). Following two washes in TE buffer (10 mM Tris-HCl and 1 mM EDTA), samples were eluted with 200 µL of fresh elution buffer (1% SDS and 0.1 M NaHCO_3_). The cross-link was reversed by adding 8 µL of NaCl (5 M) and 2 µ L of EDTA (0.5 M) to the samples and incubating overnight at 65 °C.

DNA was purified using a High Pure PCR Template Preparation Kit (Roche) and analyzed by Q-PCR.

Antibodies used: rabbit polyclonal anti-Myc (Cell Signaling 9402), rabbit polyclonal IgG control isotype (Cell Signaling 39005), or mouse monoclonal anti-GAL4 (Santa Cruz sc-510).

The following region was amplified:

**Table:** 

CD47 promoter –343/–227	5′-GACAGGACGTGACCTGGAAG-3′ 5′-GTCGCAGGCTCCAGACC-3′

## Supplementary information

Supplementary Figures

Supplementary Materials and Methods
