# Supplementary material for: Regulation of senescence escape by TSP1 and CD47 following chemotherapy treatment
Source: Cell Death Dis. 2019 Feb 27;10(3):199. doi: 10.1038/s41419-019-1406-7 (PMC6393582; doi:10.1038/s41419-019-1406-7)
Supplement: Supplementary file 1 — Supplementary Figures [file 41419_2019_1406_MOESM1_ESM.pdf]

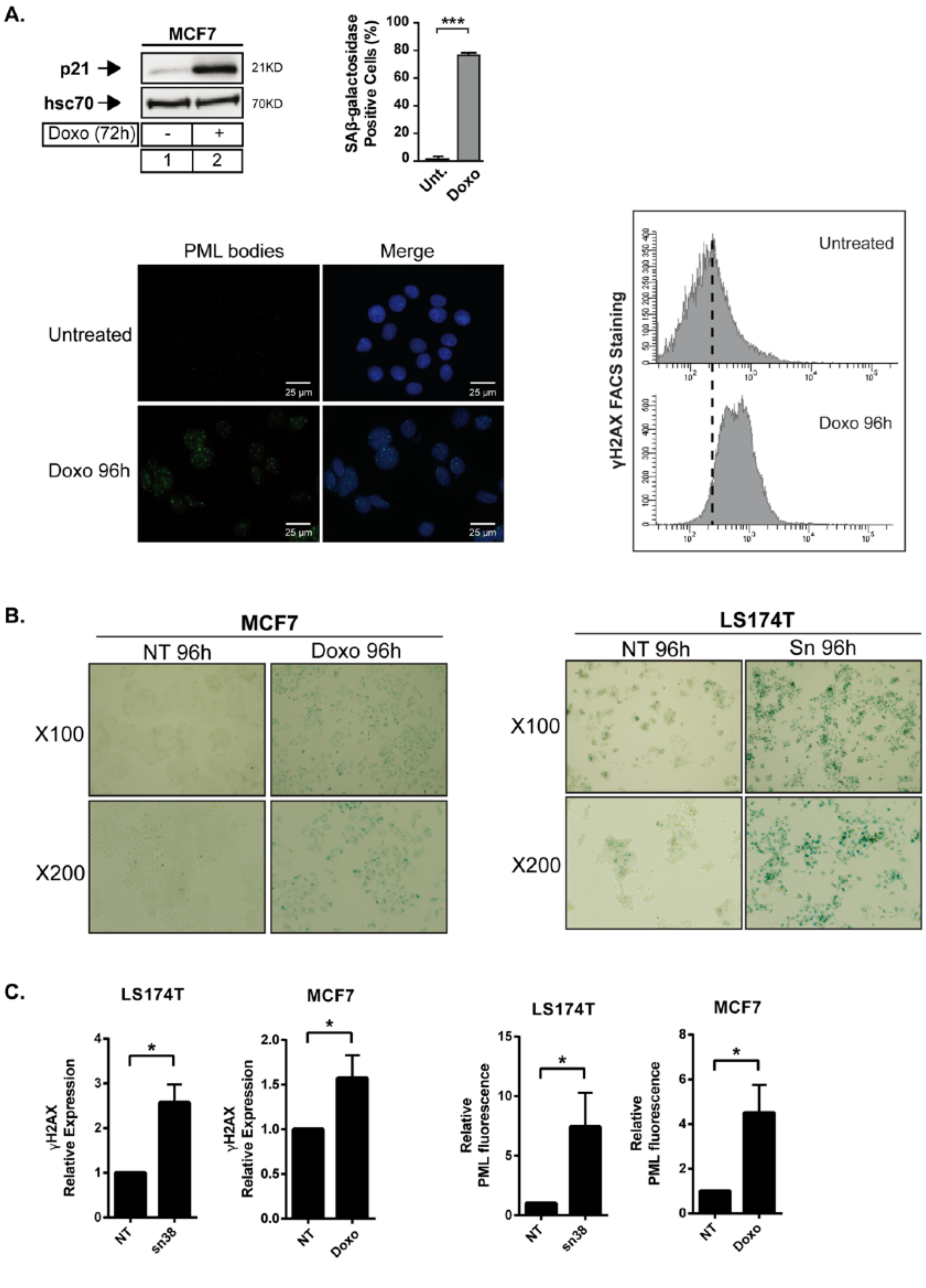

Supplementary Figure 1

Supplementary Figure 1 Legend

A. Following doxorubicin treatment of MCF7 cells (25 ng/ml), senescence was detected by the evaluation of p21waf1 expression, β-galactosidase, PML bodies and flow cytometry analysis of ɣ-H2Ax staining (n=4+/- sd, Kolmogorov-Smirnov test, *** = p<0,001).

B. Representative images of β-galactosidase staining in MCF7 and LS174T cells.

C. Quantification of ɣ-H2Ax and PML bodies staining (n=4 +/- sd, Kolmogorov-Smirnov test, * = p<0,05).

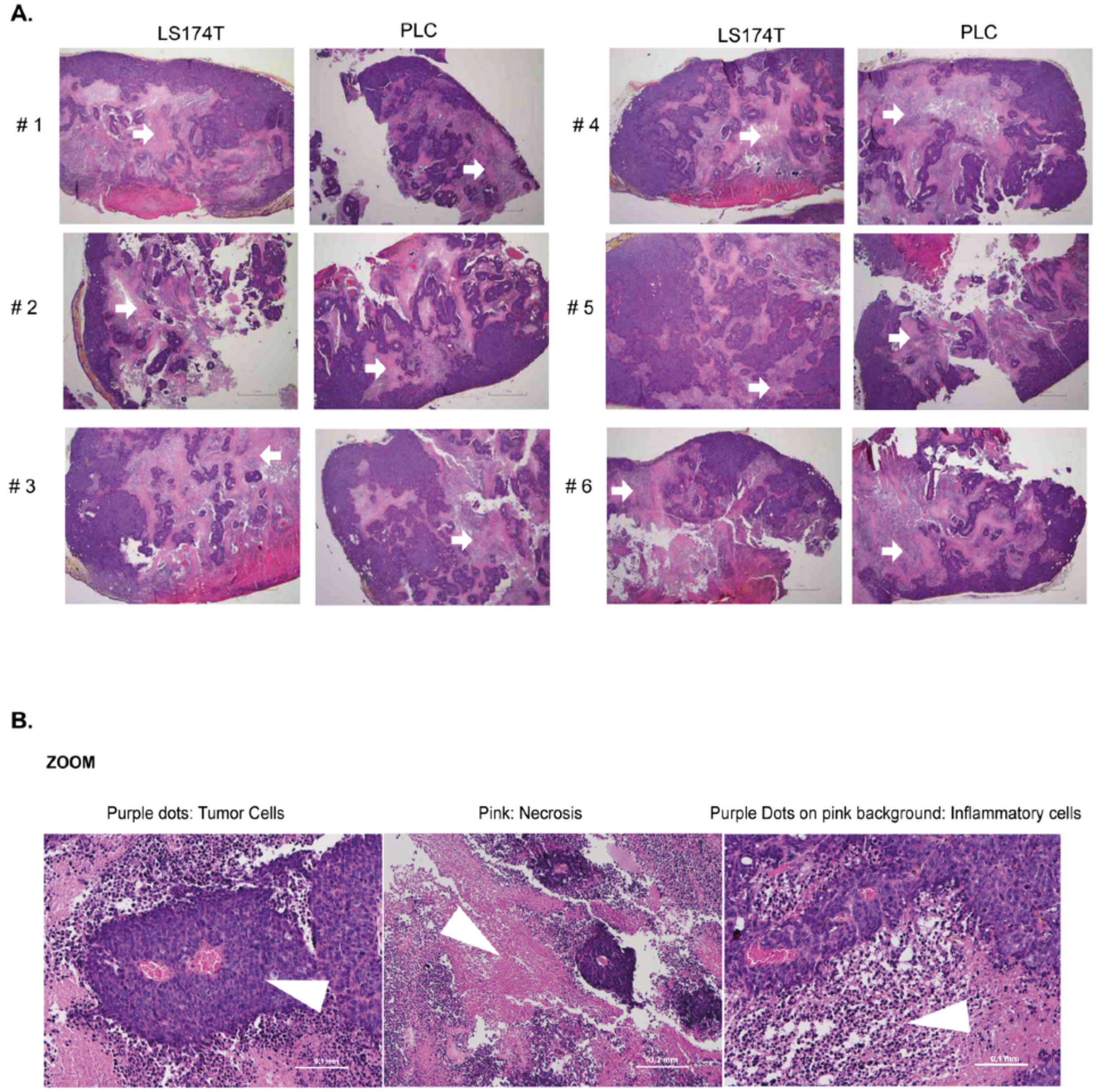

Supplementary Figure 2

Supplementary Figure 2 Legend

Parental and emergent LS174T cells were injected subcutaneously in NOD/SCID mice. Tumors were recovered and histological specimen of LS174T or PLC tumors were stained (n=6). Area of necrosis are identified by hematoxylin and eosin staining, the purple dots correspond to tumor cells, pink regions correspond to necrosis, purple dots on pink regions correspond to inflammatory cells. In our experiments, the necrotic area is generally composed of dead cells and abnormal eosinophils as shown in one illustrative zoomed picture (B). The percentage of necrosis corresponds to the percentage of the necrotic region as compared to the total surface.

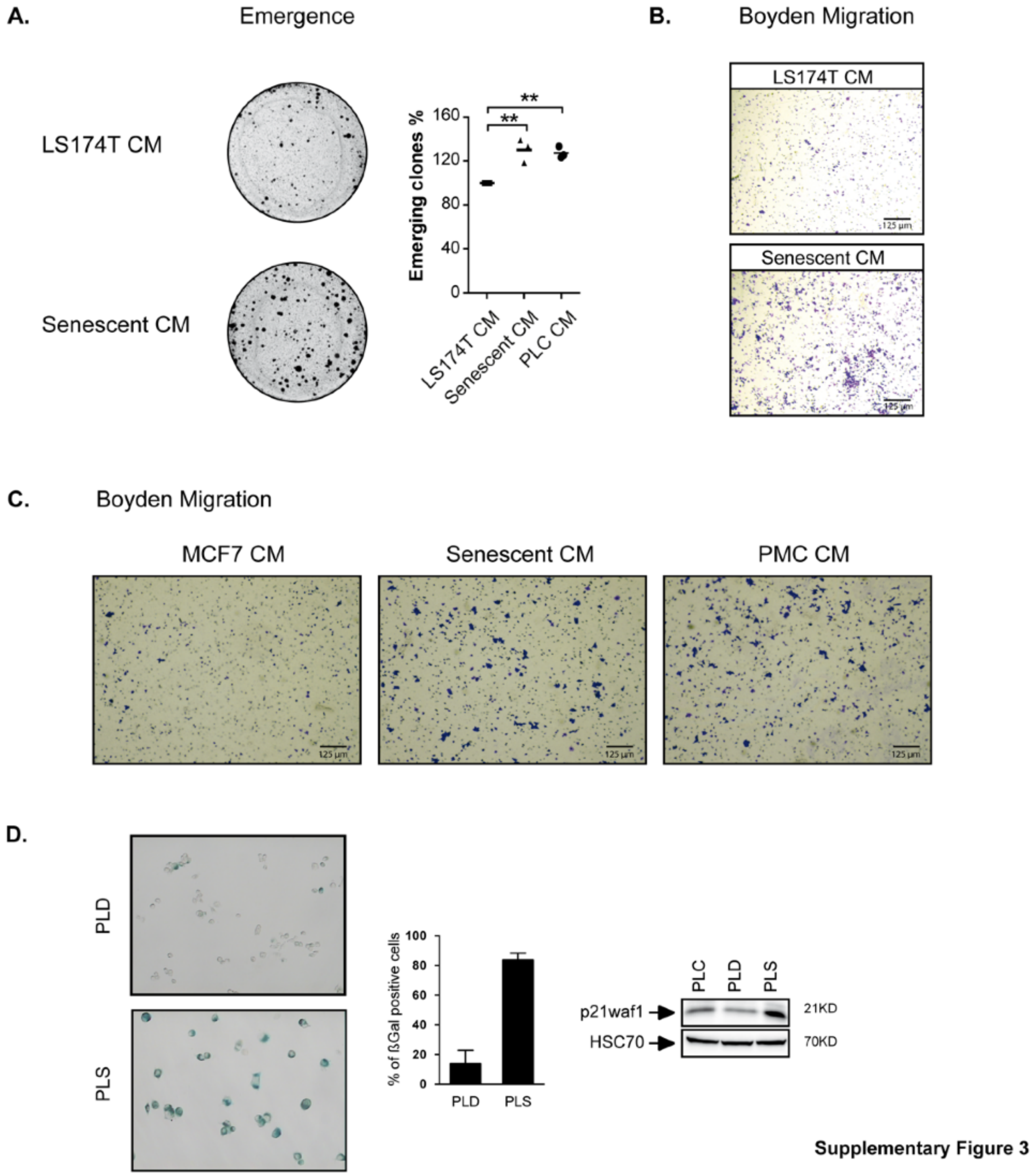

Supplementary Figure 3 Legend

A. Senescent cells were generated by treating LS174T cells with sn38 for 96h. Conditioned media (CM) were collected after 24h of serum starvation. Cell emergence was induced after treatment for 7 days using conditioned medium supplemented with 10% FBS. Clones were then counted using crystal violet staining (n=3).

B. Migration assays were performed using Boyden inserts. Conditioned media obtained from LS174T parental or senescent cells stimulated with 10%FBS for 48h and supplemented with 3% FBS were placed at the well bottom. After 72h, migrating cells were stained with crystal violet (one image representative of three experiments).

C. Migration assays were performed using Boyden inserts. Conditioned media obtained from MCF7 parental, senescent or emergent cells stimulated with 10% FBS for 48h and supplemented with 3% FBS were placed at the well bottom. After 48h, migrating cells were stained with crystal violet (one image representative of three experiments).

D. Following emergence of LS174T cells, PLD and PLS cells were cell sorted and p21waf1 expression and β-galactosidase staining were evaluated (n=3, one representative image).

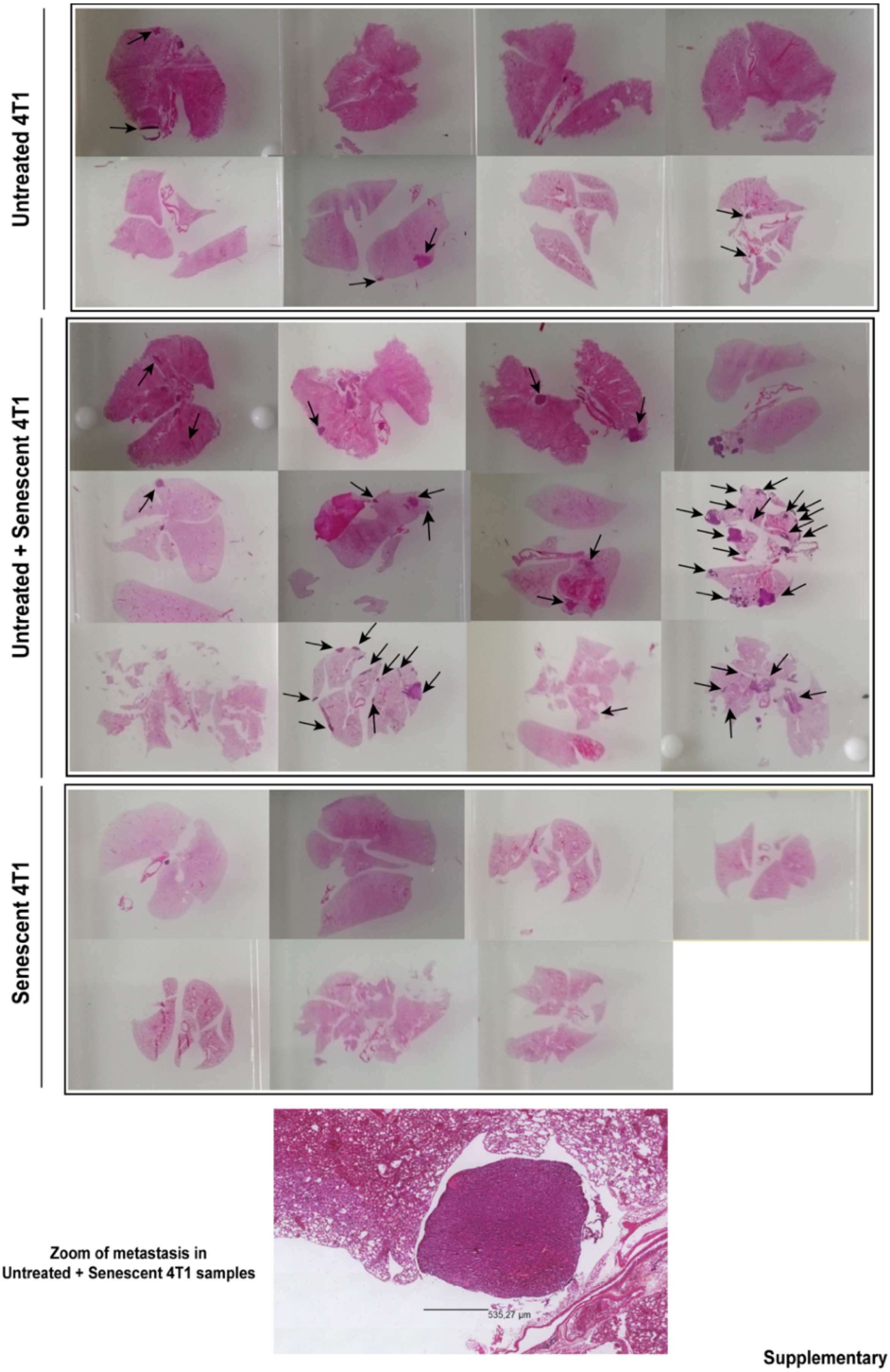

Supplementary Figure 4

Supplementary Figure 4 Legend

Representative images of lung metastasis.

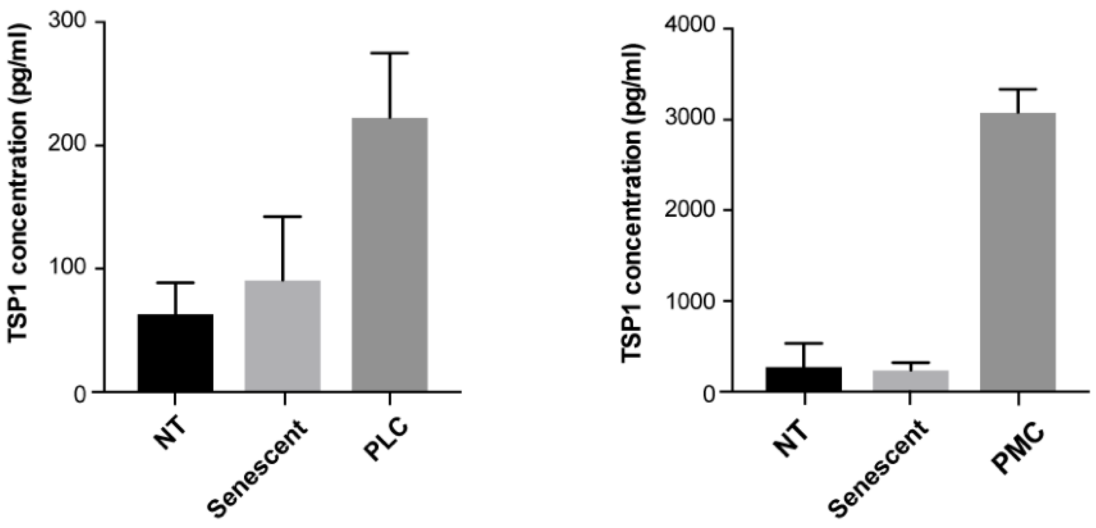

Supplementary Figure 5

Supplementary Figure 5 Legend

Following senescence induction by sn38 or doxorubicin, emergent cells were generated from LS174T or MCF7 cells by adding 10% serum for 7 days. Cells were then serum-starved for 24h and supernatants were recovered. TSP1 concentration was evaluated by ELISA analysis (n=3+/-sd).

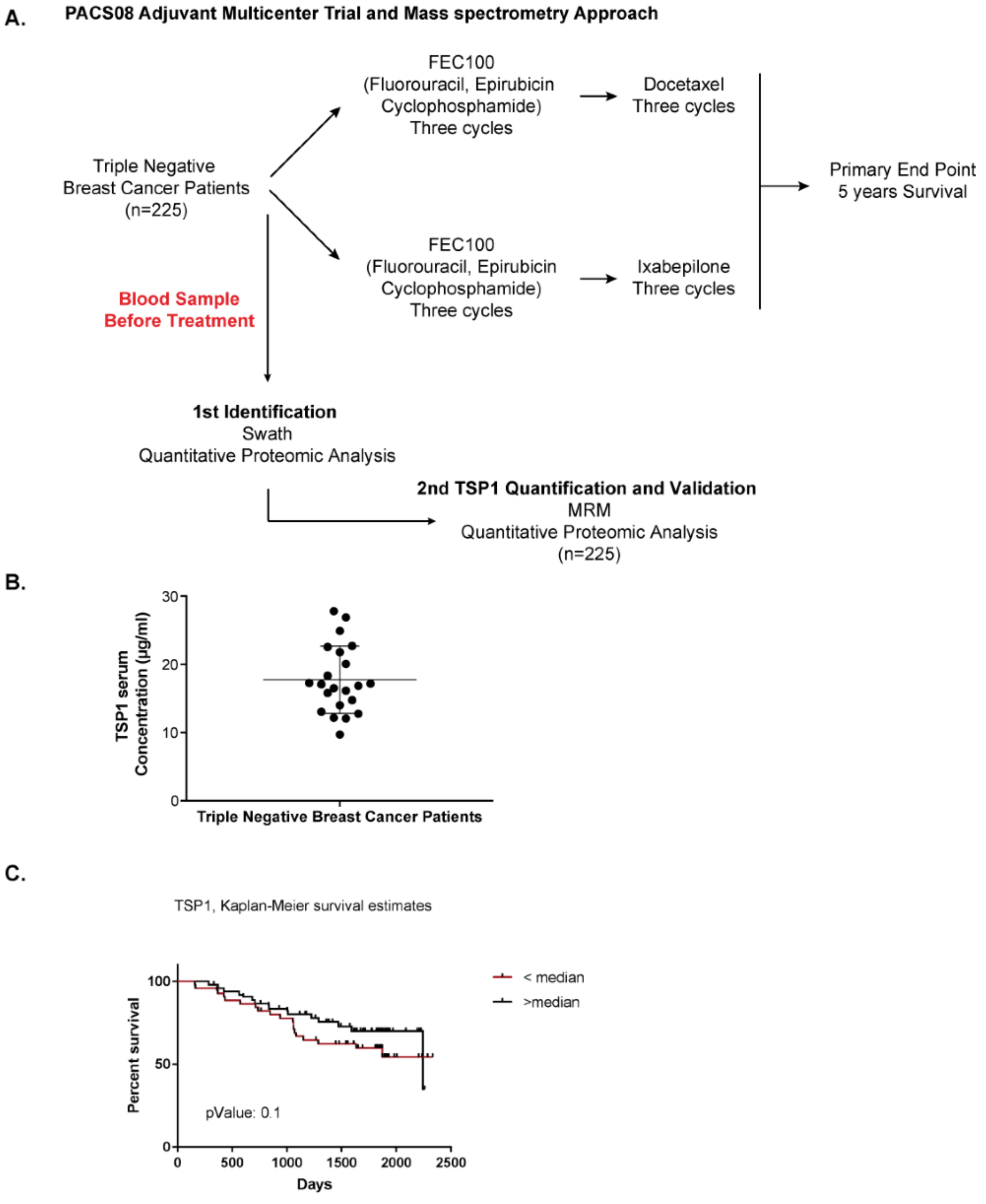

Supplementary Figure 6

Supplementary Figure 6 Legend

A. Description of the PACS08 clinical trial, blood collection and mass spectrometry approach. See clinical trial ID: NCT00630032. Relapse was defined as: a local or regional relapse; a metastatic relapse, a contralateral breast cancer, or death from any cause. Note that blood samples were obtained before chemotherapy treatment.
B. ELISA analysis of TSP1 seric levels in patients that suffer from triple negative breast cancers (n=22).
C. Kaplan Meier analysis of the link between survival and TSP1 expression (n=225).

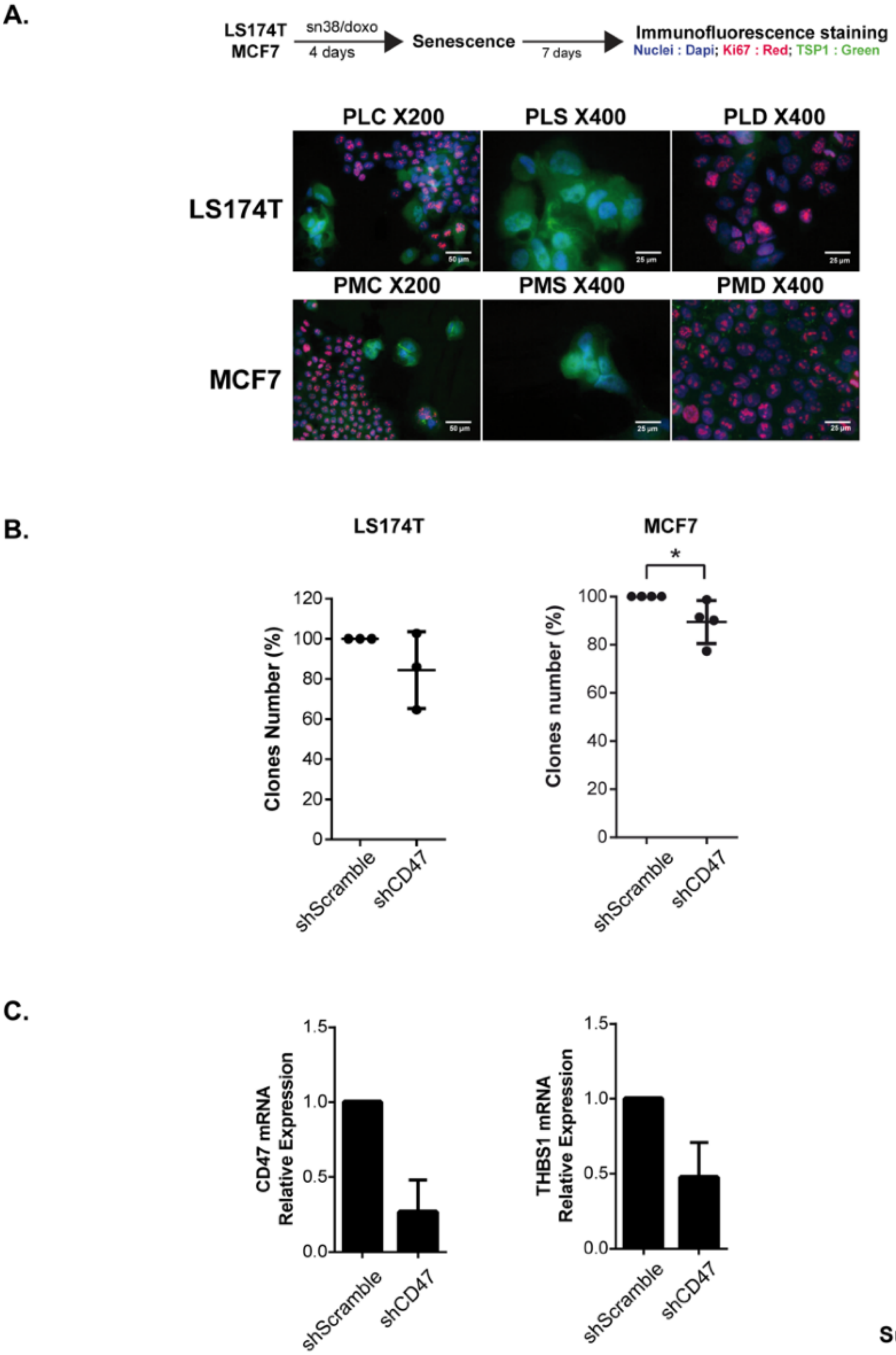

Supplementary Figure 7

Supplementary Figure 7 Legend

A. Senescence was induced in LS174T or MCF7 cells and TSP1 expression was evaluated on emergent cells by immunofluorescence, either on the total population (left) or on senescent (middle) and dividing (right) subpopulations. Ki67 staining identifies the clones that have restarted proliferation in the middle of senescent cells (n=3, one representative image).

B. Cells have been infected with a lentivirus expressing an shRNA directed against CD47 or a non targeting control. Clonogenic tests were then performed and cell growth was analyzed after 8 days (n=3 for LS174T, n=4 for MCF7 +/- sd. Kolmogorov-Smirnov test, *= p<0,05).

C. LS174T cells have been infected with a lentivirus expressing an shRNA directed against CD47 or a non targeting control. Senescence was then induced for 4 days, 10% serum was added to start emergence and mRNAs were then extracted after two days to evaluate the expression of CD47 and TSP1 by RT-QPCR (n=3 +/- sd).

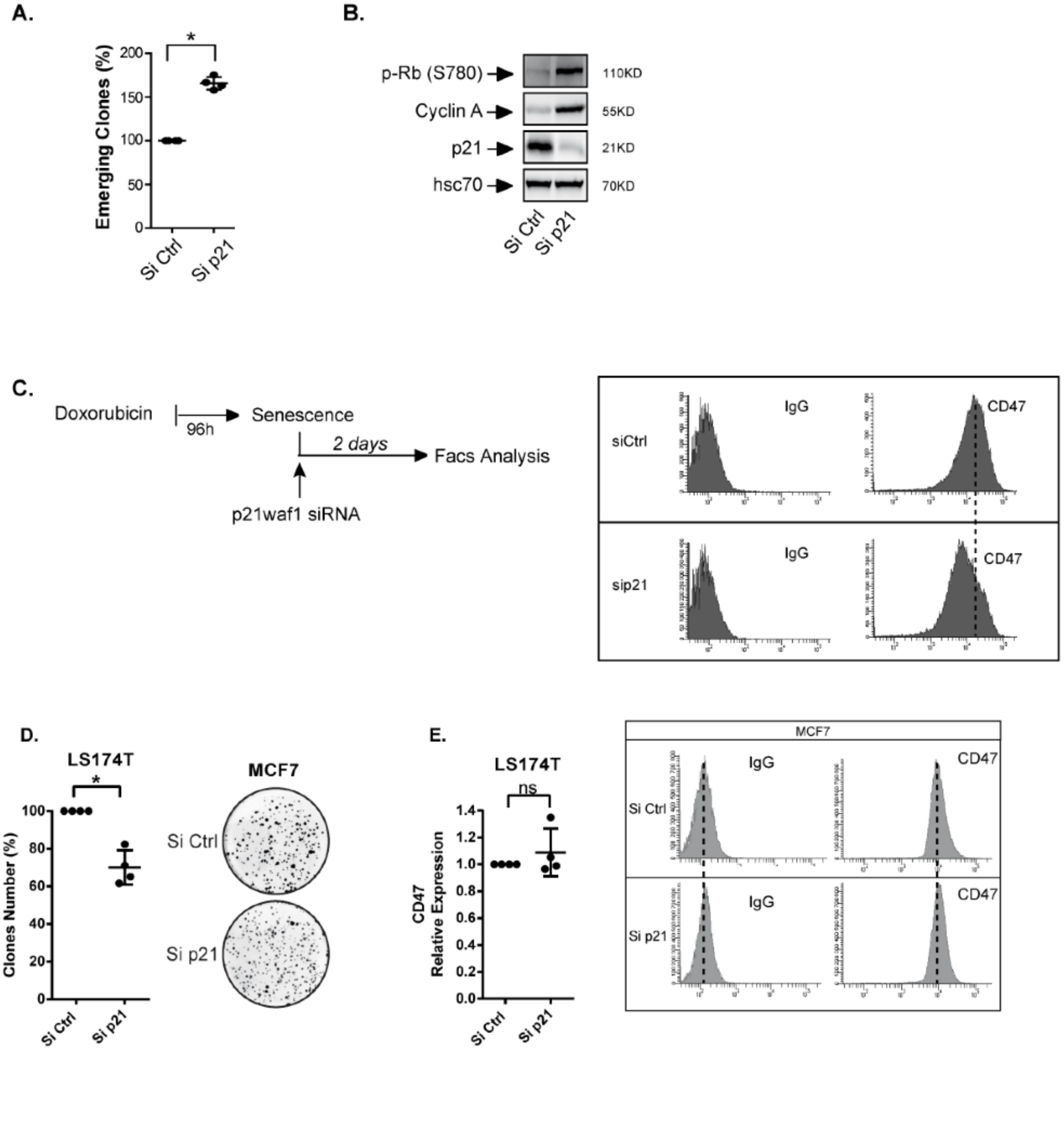

Supplementary Figure 8

Supplementary Figure 8

A. Senescence was induced in MCF7 and after 96h cells were transfected with control siRNA or siRNA directed against p21waf1. 10% FBS was added after two days to allow cell emergence (n=4).

B. MCF7 cells were treated as above and cell extracts were recovered two days after p21waf1 inactivation by siRNA. The expression of the indicated proteins was analyzed by western blot (n=4).

C. MCF7 cells were treated as above to induce senescence and after 4 days, cells were transfected with control siRNA or siRNA directed against p21waf1. CD47 expression was analyzed by flow cytometry after two days (n=4).

D, E. Growing cells were transfected with control siRNA or siRNA directed against p21waf1. Clonogenic tests were then performed and cell growth was analyzed after 8 days (D) and the expression of CD47 was analyzed in growing cells in parallel by flow cytometry (n=4 +/- sd, Kolmogorov-Smirnov test, *= p<0,05 one representative image is shown).

A.

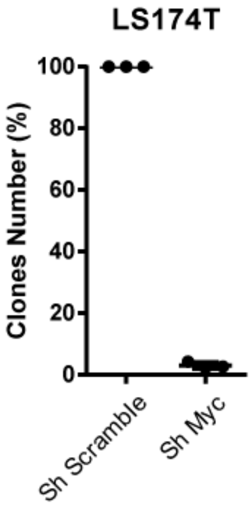

B.

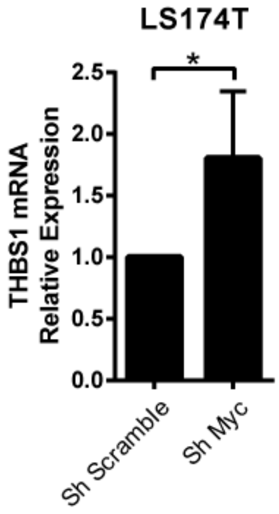

Supplementary Figure 9

Supplementary Figure 9 Legend

A. LS174T cells have been infected with a lentivirus expressing an shRNA directed against Myc or a non targeting control. Clonogenic tests were then performed and cell growth was analyzed after 8 days (n=3 +/- sd).

B. In parallel, mRNAs were extracted and the expression of THBS1 was evaluated by RT-QPCR (n=4 +/- sd. Kolmogorov-Smirnov test, *= p<0,05).

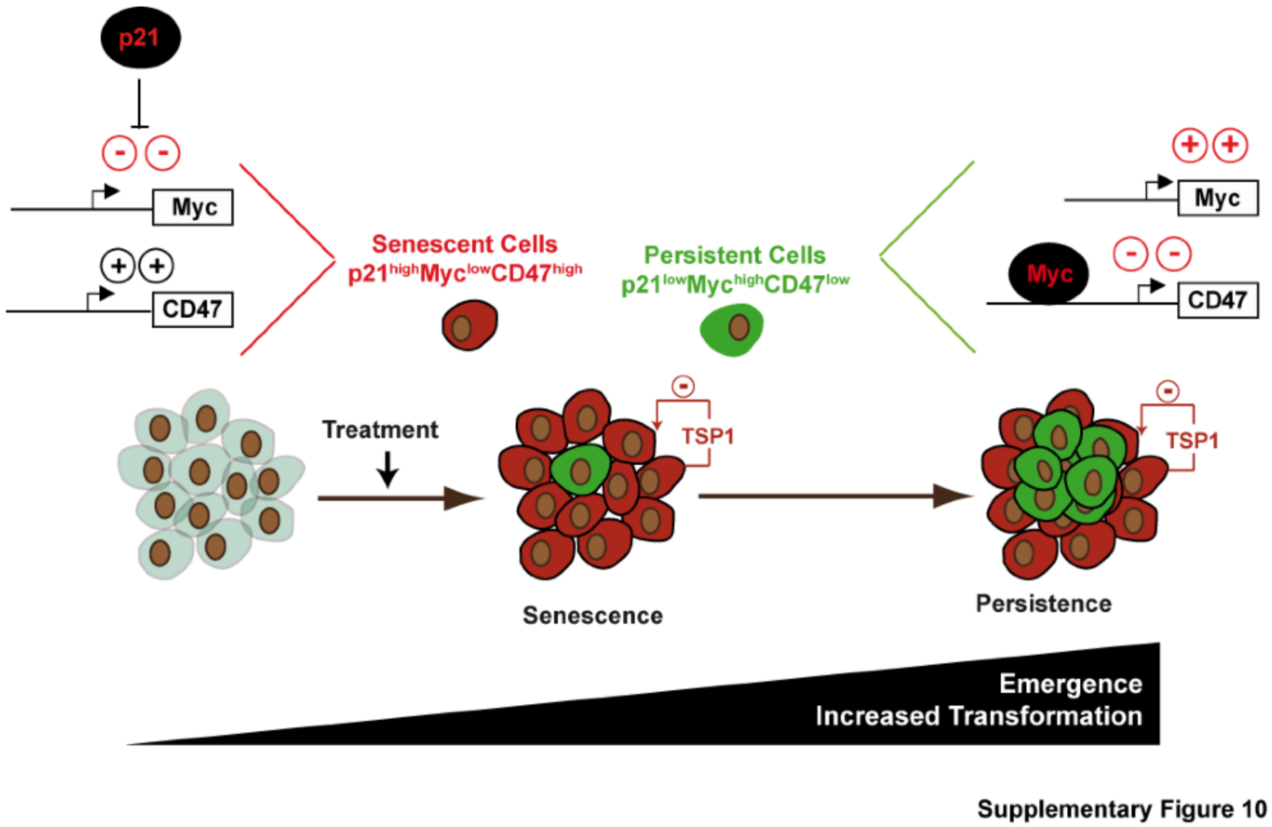

Supplementary Figure 10: Summary Model
