# Supplementary material for: Regulation of senescence escape by TSP1 and CD47 following chemotherapy treatment
Source: Cell Death Dis. 2019 Feb 27;10(3):199. doi: 10.1038/s41419-019-1406-7 (PMC6393582; doi:10.1038/s41419-019-1406-7)
Supplement: Supplementary file 2 — Supplementary Materials and Methods [file 41419_2019_1406_MOESM2_ESM.docx]

**Mass spectrometry analysis.**

*Clinical trial.* The PACS08 study (Clinical trials ID: NCT00630032) was approved by the local ethics committee and performed in accordance with the Declaration of Helsinki. Signed informed consent was obtained from all participants before study entry. Relapse was defined as: a local or regional relapse; a metastatic relapse; a contralateral breast cancer; or death from any cause.

*Immunoaffinity depletion of high-abundance proteins.*The 14 most highly abundant proteins were removed from the serum, using antibody-based depletion with a Human 14 Multiple Affinity Removal System, MARS-Hu 14 (Agilent Technologies, Santa Clara, CA, USA). Two different spin cartridges were used to deplete 2 times 10 µl of 0.22 µm-filtered plasma. This process required 2 buffers, A and B (Agilent Technologies). The pH 7.4 phosphate salt-containing buffer A was used for the equilibration, loading and washing steps. Flow-through fractions containing low-abundance proteins were collected and stored at -80°C until they were ready for analysis. A pH 2.5 urea buffer B was used for elution of the bound, highly abundant proteins from the cartridge. The experiment was conducted at room temperature according to the protocol supplied by the manufacturer.

*Creation of the spectral library*. In order to build the spectral library, peptide solutions of several protein samples were analysed by a shotgun approach by micro-LC–MS/MS. Five pooled samples of breast, colorectal and blood tissus were prepared to obtain a good representation of the peptides. Each sample were fractionated by offgel fractionator in 24 fractions. Each fraction was separated into a micro-LC system Ekspert nLC400 (Eksigent, Dublin, CA, USA) using a ChromXP C18CL column (0.3 mm × 15 cm, 3 μm, 120 Å) (Eksigent) at a flow rate of 5 μL/min. Water and ACN, both containing 0.1% formic acid, were used as solvents A and B, respectively. The following gradient of solvent B was used: 0 to 5 min 5% B, 5 to 125 min 5% to 35% B, then 9 min at 95% B, and finally 9 min at 5% B for column equilibration. As the peptides eluted, they were directly injected into a hybrid quadrupole-TOF mass spectrometer Triple TOF 5600 + (Sciex, Redwood City, CA, USA) operated with a ‘top 30’ data-dependent acquisition system using positive ion mode. The acquisition mode consisted of a 250 ms survey MS scan from 400 to 1250 m/z, followed by an MS/MS scan from 200 to 1500 m/z (75 ms acquisition time, 350 mDa mass tolerance, rolling collision energy) of the top 30 precursor ions from the survey scan.

The peptide and protein identifications were performed using Protein Pilot software (version 5.0, Sciex) with a human Swiss-Prot/TrEMBL concatenated target-reverse decoy database (downloaded in March 2016) containing 142,441 target human protein sequences, specifying MMTS as Cys alkylation. The false discovery rate (FDR) was set to 0.01 for both peptides and proteins. The MS/MS spectra of the identified peptides were then used to generate the spectral library for SWATH peak extraction using the add-in for PeakView Software (version 2.2, Sciex) MS/MS^ALL^ with SWATH Acquisition MicroApp (version 2.0, Sciex). Peptides with a confidence score above 99% as obtained from Protein Pilot database search were included in the spectral library.

*Relative quantification by SWATH acquisition*. 225 blood samples (64 from Relapse group and from 161 No-Relapse group) were analysed using a DIA method. Each sample (5 μg) was analysed using the LC–MS equipment and LC gradient described above, using a SWATH-MS acquisition method. The method consisted of repeating the whole gradient cycle, which consisted of the acquisition of 35 TOF MS/MS scans of overlapping sequential precursor isolation windows (25 m/z isolation width, 1 m/z overlap, high sensitivity mode) covering the 400 to 1250 m/z mass range, with a previous MS scan for each cycle. The accumulation time was 50 ms for the MS scan (from 400 to 1250 m/z) and 100 ms for the product ion scan (230 to 1500 m/z), thus making a 3.5 s total cycle time.

*Data analysis*. The targeted data extraction of the SWATH runs was performed by PeakView using the MS/MS^ALL^ with SWATH Acquisition MicroApp. PeakView processed the data using the spectral library created from the shotgun data. Up to ten peptides per protein and seven fragments per peptide were selected, based on signal intensity; any shared and modified peptides were excluded from the extraction. The retention times from the peptides that were selected for each protein were realigned in each run according to iRT peptides (Biognosys AG, Schlieren/Zürich, Switzerland) spiked in each sample and eluting along the whole time axis; the extracted ion chromatograms were generated for each selected fragment ion. PeakView computed a score and an FDR for each assigned peptide using chromatographic and spectra components; only peptides with an FDR of less than 5% were used for protein quantitation. The peak areas for peptides were obtained by summing the peak areas of the corresponding fragment ions; protein quantitation was calculated by summing the peak areas of the corresponding peptides. MarkerView (version 1.2, Sciex) was used for signal normalisation, and differential abundance was tested by applying a t-test at the protein level.

*Proteomic Analysis with MRM–MS*. The MRM assay was performed on a Triple TOF 5600 mass spectrometer (AB SCIEX, Framingham, MA) equipped with a micro-LC system Ekspert nLC400 (Eksigent, Dublin, CA, USA) using a ChromXP C18CL column (0.3 mm × 15 cm, 3 μm, 120 Å) (Eksigent). The peptides prepared from the sera were eluted with a nonlinear gradient program at 5 μL/min. The mobile phases consisted of solvent A, water with 0.1% aqueous formic acid, and solvent B, 100% acetonitrile with 0.1% formic acid. Peptides were separated and eluted with a gradient of 5–35% solvent B for 40 min followed by 35%–90% solvent B for 5 min. The MS parameters for all of the MRM experiments were set as ionspray voltage (IS), 2500 V; curtain gas (CUR), 35.00; ion source gas1 (GS1), 20.00; collision gas (CAD), high; interface heater temperature (IHT), 150; declustering potential (DP), 100.00; entrance potential (EP), 10.00; Q1 and Q3, unit resolution.
The MRM methods and raw data were processed by Skyline,(16) an open-source software (<http://skyline.maccosslab.org>/), in which the modified mProphet scoring algorithm was implemented for MRM peak selection.(17, 18) The transitions extracted from SWATH MS were used as MS/MS spectral library to select peptides and transitions for the MRM assays. After data acquisition, all raw data were imported into Skyline. Skyline used a scoring model for peak selection with q-values under 0.05 (corresponding to an estimated false discovery rate of 0.05 or lower). For peptides with no detected peak met this criterion, Skyline did not identify any peaks. MultiQuant 3.0 (Sciex) was also employed to evaluate protein abundances in MRM experiments.
